# Antibiotic Potentiators Against Multidrug-Resistant Bacteria: Discovery, Development, and Clinical Relevance

**DOI:** 10.3389/fmicb.2022.887251

**Published:** 2022-07-01

**Authors:** Meenal Chawla, Jyoti Verma, Rashi Gupta, Bhabatosh Das

**Affiliations:** ^1^Molecular Genetics Laboratory, Infection and Immunology Division, Translational Health Science and Technology Institute, Faridabad, India; ^2^Department of Microbiology, Institute of Home Economics, University of Delhi, New Delhi, India

**Keywords:** antimicrobial resistance (AMR), mobile genetic elements (MGEs), reversal of antibiotic resistance, antibiotic potentiators, multidrug resistance, pathogens

## Abstract

Antimicrobial resistance in clinically important microbes has emerged as an unmet challenge in global health. Extensively drug-resistant bacterial pathogens have cropped up lately defying the action of even the last resort of antibiotics. This has led to a huge burden in the health sectors and increased morbidity and mortality rate across the world. The dwindling antibiotic discovery pipeline and rampant usage of antibiotics has set the alarming bells necessitating immediate actions to combat this looming threat. Various alternatives to discovery of new antibiotics are gaining attention such as reversing the antibiotic resistance and hence reviving the arsenal of antibiotics in hand. Antibiotic resistance reversal is mainly targeted against the antibiotic resistance mechanisms, which potentiates the effective action of the antibiotic. Such compounds are referred to as resistance breakers or antibiotic adjuvants/potentiators that work in conjunction with antibiotics. Many studies have been conducted for the identification of compounds, which decrease the permeability barrier, expression of efflux pumps and the resistance encoding enzymes. Compounds targeting the stability, inheritance and dissemination of the mobile genetic elements linked with the resistance genes are also potential candidates to curb antibiotic resistance. In pursuit of such compounds various natural sources and synthetic compounds have been harnessed. The activities of a considerable number of compounds seem promising and are currently at various phases of clinical trials. This review recapitulates all the studies pertaining to the use of antibiotic potentiators for the reversal of antibiotic resistance and what the future beholds for their usage in clinical settings.

## Introduction

Antibiotics were one of history’s greatest medical breakthroughs, and their discovery altered the twentieth century. However, bacteria rapidly adopted a means to survive and neutralize the actions of antibiotics. Increased manufacture of new antibiotics with improved activity and innovative methods of action was used to combat the resistance crisis. However, the irrational and improper use of antibiotics has led to the emergence of multiple drug-resistant pathogens, which are spreading at dangerously higher levels. The new resistance attributes are circulating at a rate faster than our ability to develop new antibiotics, threatening the ability to fight infectious diseases, surgery and successful organ transplantations. Moreover, there is a lack of development of antimicrobial drugs by pharmaceutical companies and the major driving factor for the same is low return on the investment (Bettiol et al., 2015). The bacterial mechanisms to resist antibiotics cannot be stopped but certainly the rate can be slowed down by judicious use of antibiotics and discover newer ways to fight bacterial infections.

Antibiotic resistance can emerge in bacterial populations in a variety of ways, including (i) the presence of inherent resistance genes in the genome, (ii) genetic mutations during replication that allow bacteria to survive antibiotic stress, (iii) gene transfer that aids in the spread of drug resistance, (iv) widespread use of antibiotics in agriculture and injudicious usage in clinical settings (Ventola, 2015). Divergent approaches are being undertaken by researchers to cope with increasing resistance, which includes (a) development of newer scaffolds, (b) improving the efficacy of existing antibiotics by chemical modification, (c) compounds with antibiotic adjuvant activity (Piddock, 2011), (d) combination drug therapy, combining drugs acting on different targets (Lee et al., 2003) or acting on single target with different dimensions (Hansen et al., 2003), (e) Bacteriophage therapy, killing the bacteriophage occupied bacterial cells (Herelle and Smith, 1922), (f) Fecal microbiota transplantation, for restoration of healthy gut commensals by infusion of stool from healthy individual (Bakken et al., 2011), and (g) Antimicrobial peptides, which possess broad immune modulatory activity (Jenssen et al., 2006).

Inhibitors with resistance reversal activity do not usually have their own antibacterial property, but administered with antibiotics to heighten the activity of antibiotics. They are also termed as chemosensitizers (McCusker et al., 2019), antibiotic adjuvants (Wright, 2016), or resistance breakers (Laws et al., 2019). Such inhibitors have attracted the attention of several researchers and some are in clinical use already such as the combination of amoxicillin, the β-lactam antibiotic and clavulanic acid, the β-lactamase inhibitor (Brown, 1986). In 2017, the FDA approved the combination of carbapenem antibiotic and β-lactamase inhibitor meropenem–vaborbactam (Vabomere) for the treatment of urinary tract infections caused by multidrug resistant gram-negative bacteria. Certain efflux pump inhibitors (EPIs) such as PaβN (phenylarginyl-β-naphthylamide), a simple naphthylamide peptide, has also been discovered and studied, however, its clinical usage has not been initiated due to toxicity issues (Lomovskaya and Bostian, 2006).

Synthetic antibiotic potentiators make up the majority of antibiotic potentiators utilized in clinical practice today. Supplementary Table 1 includes a list of recent synthetic potentiators studied and their clinical state. Natural inhibitors with reversal activity are being explored due to the tremendous potential of resistance reversal and the concern of toxicity associated with manufactured drugs. This review article will discuss the natural antibiotic potentiators that have been investigated to date for the reversal of antibiotic resistance, as well as their varied antimicrobial resistance reversal mechanisms.

## Antimicrobial Resistance and Its Mechanisms

When a bacterial population is no longer susceptible to an antimicrobial agent then the bacteria is termed as resistant to that antimicrobial. Bacterial species can either have intrinsic ability to resist antimicrobial agents or it can acquire resistance genes from other bacterial species (Paul et al., 2022). Intrinsic resistance is because of the inherent functional or structural characteristics of bacteria that allow tolerance to a particular antimicrobial agent (Cox and Wright, 2013). Horizontal Gene Transfer (HGT) enables the acquisition of resistance genes. We have discussed below in detail the different mobile genetic elements carrying antimicrobial resistance genes in bacterial species. The common mechanisms of antimicrobial resistance involve inactivation or modification of drug, limiting the drug uptake, drug target modifications, and decreasing the active concentration of drug inside the cell through drug efflux.

### Enzymatic Modifications and Degradation of the Antibiotics

Different enzymes synthesized by the bacterial group that either removes or adds a specific moiety to the antibiotic molecule achieve the modification of drugs. The biochemical reactions catalyzed by such enzymes involve (a) phosphorylation (chloramphenicol, aminoglycosides), (b) acetylation (chloramphenicol, aminoglycosides, streptogramins), and (c) adenylation (lincosamides, aminoglycosides). Because of these structural changes in drug molecules, it is no longer able to bind to its specific target resulting in drug tolerance. The drug inactivation is also achieved by enzymes that degrade the drug molecule and a classic example of that is β-lactamases that break the amide bond of the ring making the β-lactam antibiotic ineffective (Munita and Arias, 2016; Reygaert et al., 2018).

### Target Site Modifications

Modifications of the drug target include point mutations in the gene encoding target site, enzymatic change of the target site, and bypassing the original site. The example of accumulating point mutation in target sites involve genes that encode DNA gyrase (*gyrA*) and topoisomerase IV (*parC*) making them resistant to fluoroquinolones that act by inhibiting DNA transcription and replication (Gorgani et al., 2009). The mechanism of macrolide resistance involves enzymatic methylation of ribosomes by enzymes encoded by *erm* (*e*rythromycin *r*ibosomal *m*ethylation) genes. Enzymes are capable of adding one or two methyl groups to an adenine residue at position A2058 of the domain V of the 23S rRNA of 50S ribosomal subunit (Leclercq, 2002). By avoiding the target location, bacteria develop novel targets that are similar but not identical to the original. Antibiotics will no longer be able to block the metabolic activity performed by newly developed comparable targets as a result of this. Novobiocin resistance is achieved by the production of the B component of DNA gyrase, while rifamycin resistance is achieved through the use of an additional resistant RNA polymerase (Blanco et al., 1984; Thiara and Cundliffe, 1988).

### Decreased Uptake of the Antibiotic

Another mechanism used by bacteria, particularly gram-negative bacteria, is to limit antimicrobial agent uptake. Gram-negative bacteria are protected from hydrophilic antibiotics such as β-lactams, tetracyclines, and certain fluoroquinolones given the existence of the outer membrane (Nikaido, 2003; Pagès et al., 2008). Gram-negative pathogen infections are harder to manage than gram-positive pathogen infections attributed to the prevalence of strong inherent drug resistance mechanisms in them (Arzanlou et al., 2017). These antibiotics penetrate through porins and mutation in the porin genes has increased their susceptibility for gram-negative bacteria (Li et al., 2012). Due to lack of penetration, vancomycin is ineffective against gram-negative bacteria. The porins are modified by change in the type of porins expressed, change in the level of their expression, or reduction of the function of porins. All of these pathways result in low-level antibiotic resistance and are consequently linked to additional mechanisms like enhanced efflux pump expression (Choi and Lee, 2019).

### Bacterial Efflux Systems

Efflux pumps are transmembrane proteins that assist bacteria to survive by allowing them to evacuate or exchange antimicrobial substances from their cells. These serve as a self-defense mechanism, pushing the antibiotic out of the cell and conferring target resistance (Jo and Ahn, 2016). These pumps has been classified into five families based upon their characteristics, small multidrug resistance (SMR) family, resistance-nodulation-division (RND) family, major facilitator superfamily (MFS), multidrug and toxic compound extrusion (MATE) family and ATP-binding cassette (ABC) superfamily (Sun et al., 2014). In Gram-positive bacteria (GPB) and Gram-negative bacteria (GNB), the organization of these efflux pumps is distinct (Blair et al., 2014). RND family efflux pumps, which have a tripartite organization and eject a wide range of antibiotics and biocides, are key contributors to intrinsic antibiotic resistance in GNB. MFS transporters, such as PmrA of *Streptococcus pneumoniae*, NorA of *S. aureus*, and EmeA of *E. faecalis*, are common in GPB and expel a wide range of antibiotics from various classes (Li et al., 2015; Schindler and Kaatz, 2016). MDR efflux pumps are chromosomally encoded in the vast majority of cases, such as NorA, NorB, MepA, and MdeA in *S. aureus*, which provide intrinsic resistance in bacteria to a wide range of antibiotics, while others are encoded on plasmids such as QacA/B in *S. aureus* or transposons such as MefA and MefB in *Streptococcus* spp. (Costa et al., 2013).

## Mobile Genetic Elements: The Carriers of Antibiotic Resistance Genes

Horizontal Gene Transfer disseminates the majority of AMR genes in bacteria, which are physically related to replicative or integrative MGEs. HGT allows rapid transfer of genetic material between bacterial populations and allows them to evolve and gain fitness traits. Conjugation, transformation, and transduction are the three primary processes of HGT (Wozniak and Waldor, 2010). The six major types of MGEs discovered in the bacteria are transposons, gene cassettes and integrons, genomic islands, plasmids, bacteriophages and integrative conjugative elements (ICEs).

### Integrative Conjugative Elements

Integrative conjugative elements are mobile genetic elements that are self-transmissible and an important facilitator of HGT among bacteria Burrus et al. (2002). Since then, many ICEs in both GPB and GNB have been found, and their part in formation of biofilm, pathogenicity, and motility has been investigated. The type IV secretion system, which is required for conjugation, is encoded by ICE, which is found integrated in the host genome. Numerous similarities exist between plasmid and ICE such as (a) they both contain genes required for their DNA transfer, (b) mechanisms of their integration and excision is similar, (c) they both can mobilize non-conjugative elements and cargo genes, which provides new benefits or phenotypes to the host cell. Integration of ICEs in the host chromosome is in a dsDNA circular form, after excision from the chromosome by the ICE-encoded site-specific integrase (Int), as a result of site-specific recombination. The first conjugative element evidenced was Tn916 in *E. faecalis*, which encodes tetracycline resistance and is transferred to other strains through conjugation. This work leads to a conclusion that like a transposon, ICE Tn916 encodes genes required for conjugative transfer and can integrate into the host chromosome (Johnson and Grossman, 2015). The well-studied, diversified and largest family of ICE carrying resistance genes for multiple antibiotics is SXT/R391 family elements and is discovered in *V. cholerae* (MO10) and *Proteus rettgeri*, but can be found in *E. coli* also. It provides resistance to streptomycin, sulfamethoxazole and trimethoprim (SXT), chloramphenicol and streptomycin. SXT/R391 family elements have the ability to mobilize neighboring sequences, including a small number of genomic islands with an oriT (Xu et al., 2018).

### Insertion Sequences and Composite Transposons

The transposons are the mobile segment of DNA that is inserted into the host DNA molecule and depends on the host DNA for its replication as it has no origin of replication. DNA-based transposons are divided based on whether the new copy is formed during transposition (replicative transposition) or the original copy moves to a new location (conservative transposition). Insertion sequences (IS) are the small MGEs that carry the transposase gene necessary for its mobility and a composite transposon carries two similar or related IS elements on either side of the resistance carrying gene which moves as a single unit. These resistance transposons can jump inter- as well as intramolecularly (Bennett, 2008). Resistance to aminoglycosides such as kanamycin, neomycin, and streptomycin is provided by the transposon Tn5. It is 5.8 kb composite transposon flanked by two inverted 1.5 kb IS50 insertion sequences (Reznikoff, 1993). Similarly, Tn*10* is of 9.3 kb length flanked by 1.4 kb inverted repeats and encodes resistance for tetracycline (Foster et al., 1981). Several examples of IS and composite transposons that carry antibiotic resistance genes (ARGs) have been reviewed (Partridge et al., 2018).

### Gene Cassettes and Integrons

The integrons are the group of MGEs, which contain components required for site-specific recombination and one or more ARGs located at the specific site. An integron retains a site, *attI*, at which gene cassettes are integrated (Stokes and Hall, 1989). These are classified as class 1, 2, and 3 integrons, each encoding for a different integrase and the members within the class contain the same integrase but different gene cassettes. Gene cassettes comprise a gene flanked by 59-base element recombination site, recognized by site-specific recombinase (IntI) encoded by integron. Gene cassettes are free, circular, non-replicating molecules that are transcribed only when captured and inserted into an integron, using an integron promoter. Gene cassettes are unlike other MGEs in a way that it doesn’t have all the functions required for their mobility (Bennett, 1999). The resistance genes for various antibiotic classes such as β-lactams - *bla*P1, P2, P3, *bla* IMP etc., genes for chloramphenicol resistance - c*at*B2, B3, B5, and *cmlA*, genes for aminoglycosides resistance like *aad*A1, *aad*A2, *aad*B, and others are carried on gene cassettes (Recchia and Hall, 1995).

### Plasmids

Plasmids are double stranded, self-transmissible and autonomously replicating DNA molecules. They are useful for the transmission of diverse ARGs because of their capacity to multiply in a variety of host organisms. Most antibiotic resistant plasmids are large, low-copy-number, consisting of a replication machinery and maintenance strategies for their persistence, partitioning mechanisms for segregating plasmids to the progeny bacteria, conjugation systems, resolvases to disentangle plasmid multimers, and plasmid addiction systems that encodes stable toxin and the unstable antidote (Sengupta and Austin, 2011). Conjugative plasmids mediate not only their own transfer but also the transfer of additional plasmids from one bacterium to another. The plasmids have been typed by their potential to hamper fertility of plasmid F (Meynell et al., 1968), by phage sensitivity (Anderson et al., 1973) and by incompatibility (Inc) typing (Datta and Hedges, 1971; Novick et al., 1976). Inc defines that two related plasmids are unable to proliferate stably in the same bacterial cells; hence, only compatible plasmids can be used as transconjugants. The first Inc groups were – (a) IncI, plasmids having type I pili susceptible to Ifl phage; (b) IncN, N3-related plasmids susceptible to IKe phage; (c) IncF, 77 plasmids having type F pili susceptible to Ff phage; and (d) IncP, RP4-related plasmids susceptible to the PRR1 phage (Datta and Hedges, 1971). The most common plasmid families found in Enterobacteriaceae are IncF, which are low copy number plasmids with a size of > 100 kb and more than one replicon for replication initiation (FII, FIA, and FIB) (Carattoli, 2013). The most frequent plasmid families carrying the ARGs have been reviewed previously (Carattoli, 2009).

## Strategies to Reverse the Antibiotic Resistance

**FIGURE 1:**
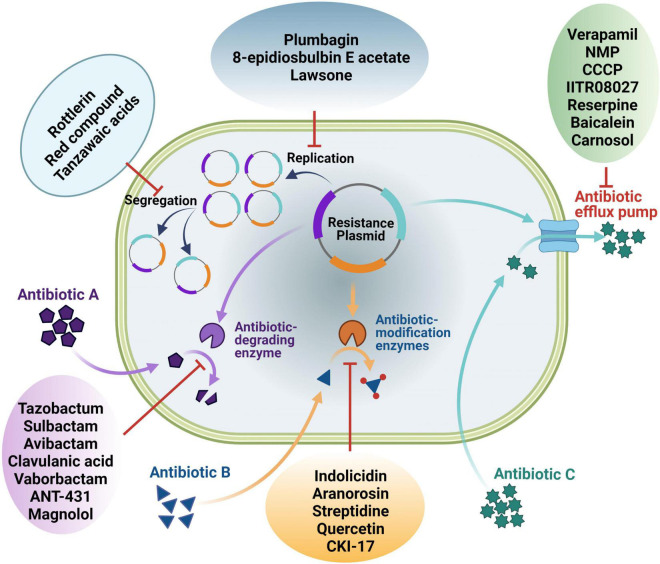
An overview of different ways of antibiotic resistance reversal by different antibiotic potentiators. Potentiators may work directly on the resistance functions or indirectly on the genetic elements linked with the resistance genes.

Widespread resistance to available antibiotics in clinically important pathogenic bacteria is currently a global challenge due to an ever-increasing number of strains that are resistant to multiple classes of antibiotics. To tackle this grave problem, the use of “antibiotic adjuvants/potentiators” in combination with antibiotics is now being exploited (González-Bello, 2017). Strategies being undertaken to reverse antibiotic resistance is discussed in this section (Figure 1).

### Inhibiting Plasmid Conjugation Mechanisms

Conjugative plasmids are one of the mediators of dissemination of ARGs. It has been studied that IncF, IncN, IncI, IncX, IncW, and IncP are eight conjugative plasmids that cover six incompatibility groups and account for more than 70% of the most common big plasmids identified from *Enterobacteriaceae*, having a broad range of conjugation efficiencies and fitness effects (Lopatkin et al., 2017). The transmission of these conjugal plasmids is extremely quick, allowing them to persist. As a result, combining conjugation inhibition with plasmid loss would be an effective technique for limiting conjugation-assisted persistence of antibiotic resistance (Lopatkin et al., 2017). The two potential compounds showing suppression of plasmid conjugal transfer have been characterized from extract of *Mallotus philippensis* (Lam.), a medicinal plant. Through one-dimensional and two-dimensional nuclear magnetic resonance (NMR), the compounds identified were rottlerin (**1)** [5,7-dihydroxy-2,2-dimethyl-6-(2,4,6-trihydroxy-3-methyl-5-acetylbenzyl)-8-cinnamoyl-1,2-chromene] and the red compound (**2**) (8-cinnamoyl-5,7-dihydroxy-2,2,6-trimethylchromene). The plasmids whose conjugative transfer inhibited were pKM101, pUB307, TP114 and R6K amongst *E. coli* at a sub-inhibitory concentration (SIC) of 100 mg/L. However, the study did not reveal the specific mechanism of plasmid conjugal transfer inhibition (Oyedemi et al., 2016; Patwardhan et al., 2018). Another study (Getino et al., 2016) identified the potential conjugation inhibitors Tanzawaic acids A (**3a**) and B (**3b**) as the candidate molecules having specific inhibition against IncFII and IncW conjugative plasmids. These compounds were observed to have lower toxicity to animal cells unlike other synthetic inhibitors.

### Plasmid Curing

Plasmid curing is the method of removing plasmids from the bacterial populations while leaving the population intact. It has the potential to remove the ARGs from the population and thus reversal of the plasmid-mediated antibiotic resistance. Plasmid cure can take place by a variety of ways, including blocking plasmid replication or interrupting plasmid segregation, leading in a steady decrease of plasmid carrying cells, or raising the fitness expenses related with plasmid carriage. Several plant-derived compounds showing plasmid curing abilities are discussed hereby.

Initially studies have identified the bioactive compound, ascorbic acid which has shown *in vitro* activity for the loss of plasmids encoding for penicillin and aminoglycoside resistance genes (Amábile Cuevas, 1988; Amábile-Cuevas et al., 1991). The conversion of covalently closed circular form into open circular form is attributed as its possible mode of action in plasmid curing (Morgan et al., 1976). However, further studies to analyze its effects under *in vivo* conditions have not been done. Another study has identified a novel plasmid-curing compound from the methanolic extract of *Dioscorea bulbifera* L. bulbs, named as 8-epidiosbulbin E acetate (**4**) (EEA – norditerpene). It displayed broad-spectrum antibiotic resistance (R-plasmids) plasmid-curing activity against clinical isolates of *E. coli, E. faecalis*, *P. aeruginosa*, and *S. sonnei* with curing efficiency of 12–48%, at the varying concentrations of EEA ranging from 25–200 μg/mL. The reference plasmids under study were RP4 of *E. coli*, pUB110 of *Bacillus subtilis*, RIP64 of *P. aeruginosa* and R136 of *S. typhi*, which were cured with efficiency of 16–64%. Also, the EEA compound did not display any cytotoxicity against MCF-7, the human breast cancer cell lines, A431 (epidermal carcinoma) and SiHa (cervical cancer) and thus making it a potential plasmid-curing compound (Shriram et al., 2008).

Further the native therapeutic plant of India, China and from countries of South Asia, *Alpinia galanga* (Linn.) Swartz (Blagden, 1937) has shown R-plasmid curing abilities against *E. coli, Salmonella typhi* and VRE (vancomycin resistant *Enterococcus faecalis)* with efficiencies of 82%, 92%, and 8% respectively, at a sub-inhibitory SIC of 400 μg/ml. The 1′-acetoxychavicol acetate (**5**) was found to be the bioactive molecule showing antibiotic resistance plasmid eliminating efficiency of 75% in *S. typhi*, 70% in *P. aeruginosa*, 32% in *E. coli* and 66% in vancomycin resistant *Enterococcus* at *SIC* ranging from 400 to 800 μg/ml. Pre-exposure with 1′-acetoxychavicol acetate of bacterial strains significantly elevated the effectiveness of antibiotics (Latha et al., 2009). The reversal activity of aqueous and methanol extracts of *Piper longum* (fruits) have also been studied against MDR clinical isolates of *E. faecalis, Staphylococcus aureus, Salmonella typhi, Shigella sonnei* with the reference plasmid containing strains of *E. coli* (RP4) and *Bacillus subtilis* (pUB110). The methanol extract had antibacterial activity against *Bacillus subtilis* with a MIC of 400 g/mL and could also reverse resistance in *S. sonnei* with a 42% cure rate, whilst the aqueous extract had good resistance reversal efficiency with a MIC of 400 g/mL against clinical strains harboring R-plasmid, *E. faecalis, S. typhi*, and *S. aureus* with cure rate of 64%, 32%, and 50% respectively. As a result, the eradication of R-plasmids could be responsible for the reversal of antibiotic resistance (Chandra et al., 2017). The aqueous extract of a fruit, *Terminalia chebula* as well exhibited anti plasmid activity against plasmid pUB110 of *B. subtilis* and pARI-815 of *S. sonnei* with curing efficiency of 100% and 44%, respectively. Further, the aqueous extract of this fruit has already been in use without any reported toxicity (Vemuri et al., 2019).

**FIGURE 2:**
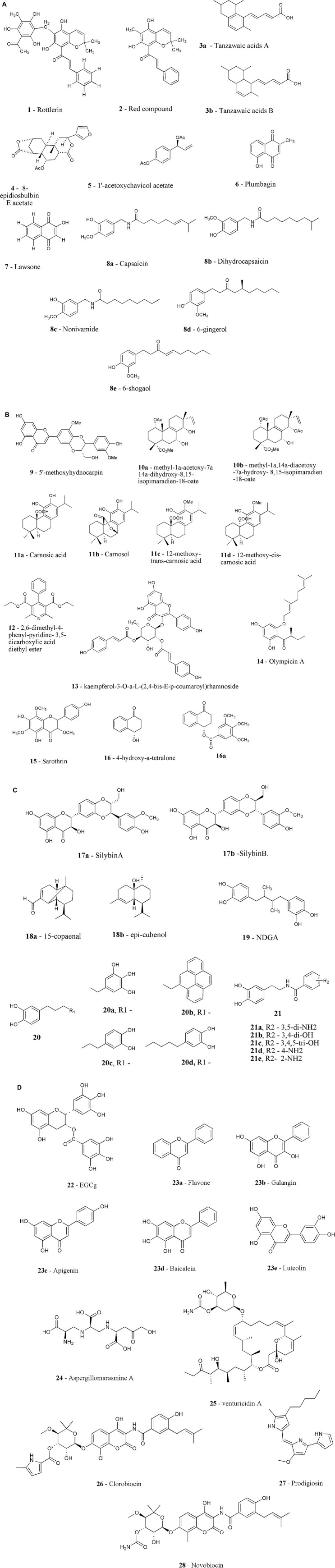
**(A)** Chemical structure of plasmid curing inhibitors (1-8e). **(B)** Chemical structure of efflux pump inhibitors (9-16a). **(C)** Chemical structure of efflux pump inhibitors (17a-21). **(D)** Chemical structure of resistance enzymes inhibitors (22-28).

The potentiality of plant root extracts as antibiotic reversal activity has been investigated as well. The plasmid curing activity determined extracts of *Plumbago auriculata*, was determined against R plasmids of several bacterial strains such as *E coli, P. aeruginosa, K. pneumoniae, S. aureus, Proteus vulgaris*, and *Enterobacter cloacae*, isolated from clinical specimens like urine and pus. Its curing efficiency in *P. aeruginosa* was 13%, 15% in *E. coli*, 32% in *P. vulgaris* and maximum in *K. pneumoniae* with 30% (Singh et al., 2018). Previously also a compound, Plumbagin (**6)** (5-hydroxy-2-methyl-l,4-naphthoquinone), obtained from the plant root of *Plumbago zeylanica* was found potent in specifically eliminating the multidrug-resistant, stringent, and conjugative plasmids from *E. coli* strains (Lakhmi et al., 1987). 2-hydroxy-1, 4-naphthoquinone, also known as lawsone (**7**), is the bioactive molecule responsible for plasmid curative activity. Lawsone has been shown to cure wild plasmids found in a number of hosts, including pBR322, pUPI281, pRK2013, R136, and pUPI282. The curing efficiency for studied plasmid ranges from 4.2% to 20%. It also prevented the transformation or conjugation of plasmid pRK2013 into *E. coli* and the compound was identified as non-toxic to mammalian cells (Patwardhan et al., 2018). Furthermore, the crude methanol extract of *Capsicum annum* has displayed good inhibition rates of 8.59% against three R-plasmids PUB307, PKM 101 and TP114. Capsaicin (**8a**) and dihydrocapsaicin (**8b**) were found as bioactive substances with anti-plasmid activities ranging from 5.03 to 31.76% against antibiotic resistance-causing plasmids such as R6K and R7K. The effect of the other capsaicin analogs nonivamide (**8c**), 6-gingerol (**8d**) and 6-shogaol (**8e)** on the plasmids were significant on PKM 101 (6.24–22.16%), TP114 (0.1–7.19%) and PUB 307 (1.22–45.63%). Capsaicin, 6-gingerol, and 6-shogaol were found to have antibacterial action against effluxing MRSA strains XU212 (TetK), RN4220 (MsrA) and SA1199B (NorA) with MIC values of 8-256 mg/L (Oyedemi et al., 2019). Chemical structures of the natural conjugal transfer inhibitors and plasmid curing compounds is shown in Figure 2A and also summarized in Table 1.

**TABLE 1:** Plant derived compounds targeting the plasmids carrying AMR genes.

S. No.	Mechanisms	Plant name	Compound/Active extracts	Targets	References
1.	Plasmid conjugation inhibitors	*Mallotus philippensis*	Rottlerin, Red compound	Plasmids pKM101, pUB307, TP114 and R6K	Oyedemi et al., 2016; Patwardhan et al., 2018
2.		*Penicillium citrinum* isolated from a *Porifera* sp.	Tanzawaic acids A and B	IncW plasmid R388 and the IncFII plasmid R100-1	Getino et al., 2016
3.	Plasmid curing		Ascorbic acid		Amábile Cuevas, 1988
4.		*Dioscorea bulbifera*	8-epidiosbulbin E acetate	RP4, pUB110, RIP64, and R136	Shriram et al., 2008
5.		*Alpinia galanga*	1′-acetoxychavicol acetate	Plasmid RP4 and pUB110	Latha et al., 2009
6.		*Piper longum*	Aqueous and methanol extracts	Plasmid RP4pUB110	Chandra et al., 2017
7.		*Terminalia chebula*	Aqueous extract	pUB110 of and pARI-815	Vemuri et al., 2019
8.		*Plumbago auriculata*,	Root extracts	R- plasmids of bacterial strains	Singh et al., 2018
9.		*Plumbago zeylanica*	Plumbagin Lawsone	Conjugative plasmids pBR322, pUPI281, pRK2013, R136, and pUPI282	Patwardhan et al., 2018
10.		*Capsicum annum*	Capsaicin and dihydrocapsaicin, nonivamide, 6-gingerol and 6-shogaol	pUB307, pKM 101 and TP114	Oyedemi et al., 2019

*The natural products derived from plants inhibit the plasmid conjugation, replication and segregation mechanisms, hence may play an important role in slowing down the dissemination of AMR genes.*

### Efflux Pump Inhibitors

The efflux pumps are responsible for antibiotic resistance by pumping the antibiotic out of the cell, decreasing their effective concentration inside the cell. Efflux pumps are more frequently identified as internal mechanisms of bacterial resistance, as genes coding for them can be found in both resistant and susceptible bacteria, and their expression is controlled at the transcriptional level (Webber, 2003). The efflux pumps over expression are the key mechanism through which bacteria shield themselves against antimicrobial medication side effects. Managing efflux pumps can thus be an effective technique for restoring antibiotic sensitivity in bacteria that have developed resistant phenotypes. There are a number of ways to abolish the efflux of antibiotics by bacteria: (i) down regulating the expression of efflux pump genes, (ii) redesigning antibiotics to be no longer recognizable as substrates, (iii) preventing the assembly of functional efflux transporters, (iv) blocking the binding of substrate to the active site, and (v) minimizing the energy mechanism providing energy to these pumps (Bhardwaj and Mohanty, 2012).

In this regard, researchers are looking for the plant extracts that manifest the inhibitory functions as these extracts will have no or low toxicity. In GNB mainly, efflux and ethidium bromide (EtBr) buildup are potential markers of efflux pump involvement (Martins et al., 2008). For this reason several studies discussed below have used such assays (Oliveira et al., 2020). Series of natural inhibitors that inhibit these traits have been discussed here.

*Callistemon citrinus*, an evergreen tree or shrub, commonly known as ‘Crimson Bottle Brush’ which is used mostly for ornamental purposes (Cock, 2012) has been a source of various bioactive compounds commonly used as antibacterial, antifungal, insecticidal and as a remedy for the treatment of dysentry, diarrhea and rheumatism. Another medicinal plant *Vernonia adoensis* is traditionally used in the Rift valley and Western Kenya for the treatment of sexually transmitted disease gonorrhea, malaria, heart, and kidney problems (Mozirandi et al., 2019). The ethanolic leaf extracts of both *C. citrinus* and *V. adoensis* from Zimbabwe depicted their potential antibacterial activities as EPIs as well as membrane permeabilizing phytochemicals (Chitemerere and Mukanganyama, 2014). The isolate of the Chinese medicinal herb *Arnebia euchroma*, isovaleryl shikonin (IVS), has exhibited antibacterial activity against *S. aureus* RN4220 (overexpress *msrA* gene), with MIC of 16 mg/L. When IVS was used with streptomycin, the MIC of streptomycin was reduced by up to 16-fold and this was because of the reduced bacterial efflux by IVS. IVS inhibited MsrA efflux pump by suppressing the expression of msrA mRNA. In the murine peritonitis/sepsis model, the IVS reduced bacterial counts in spleen, peritoneal, and liver tissue synergistically with streptomycin and extended mice survival to 7 days. The acute toxicity of IVS was investigated, and the 50% fatal dosage (LD_50_) of IVS in mice after a single exposure was found to be 2.584 g/kg (He et al., 2019).

Considerable number of studies conducted in search of EPI inhibitors have shown to reverse NorA mediated resistance in bacteria as their mechanism of action. As mentioned, NorA is a chromosomally encoded MDR efflux pump. These MDR efflux pumps could be a good place to start looking for inhibitors. Clinically significant efflux pump members like NorA belong to the MFS EPIs family found in *S. aureus* and overexpressed in 43% of all the *S. aureus* strains (Sun et al., 2014; Astolfi et al., 2017). Studies conducted involve evaluation of various plant species from the family Berberidaceae, which produce a common component, alkaloid berberine which possess weak antibiotic properties because of its efflux from NorA pump of *S. aureus*. It was proposed that certain NorA MDR inhibitors accompany berberine in species *Berberis repens, B. fremontii*, and *B. aquifolia*. The inhibitor identified was 5*-methoxyhydnocarpin (**9**) (5*-MHC), which don’t possess its own antibiotic activity but enhance activity of MDR substrates and berberine. 5*-MHC completely inhibited the efflux of berberine and EtBr from *S. aureus* cells as their level increases in cells in the presence of 5*-MHC (Stermitz et al., 2000). Another plant, *Lycopus europaeus* found in river and canal banks of Britain, commonly known as Gipsywort have anti-thyreotropic and anti-gonadotropic activities. Two isopimarane diterpenes extracts from this has been isolated, namely methyl-1α-acetoxy-7α 14α-dihydroxy-8,15-isopimaradien-18-oate (**10a**) and methyl-1α, 14α-diacetoxy-7α-hydroxy- 8,15-isopimaradien-18-oate (**10b**). These compounds and four other diterpenes extracted previously (Hussein and Rodríguez, 2000) were tested for antibacterial and resistance reversal activity against *S. aureus* strains with the Nor(A), Tet(K) and Msr(A) multidrug efflux pumps. No compounds displayed any antibacterial activity at the highest tested concentration of 512 μg/ml but when combined with erythromycin and tetracycline antibiotics, the twofold decrease in MICs of these antibiotics was observed against IS-58 and RN4220 strains of *S. aureus* that were resistant to these antibiotics because of the occurrence of Tet(K) (tetracycline resistance) and Msr(A) (macrolide resistance) efflux pump, respectively (Gibbons et al., 2003).

Another group has also evaluated the effect of *Rosmarinus officinalis* L. as EPI. It is commonly named as rosemary, a popular herb of many western countries. It offers a wide range of medicinal properties including antibacterial (Campo et al., 2000) and antioxidant properties (Ozcan, 2003). Its effects as an efflux inhibitor in *S. aureus* strains expressing multidrug efflux pumps, MsrA and TetK was studied. The known abietane diterpenes carnosic acid (**11a**), carnosol (**11b**), and 12-methoxy-*trans*-carnosic acid (**11c**), as well as the *cis*-form 12-methoxy-*cis*-carnosic acid, were identified using bioassay-guided fractionation of its chloroform extract (**11d**). Their antibacterial properties were assessed and MICs ranging from 16 to 64 μg/ml were found. Compounds **11a** and **11b**, when added to the growth medium at 10 μg/ml, increased the activity of erythromycin against an erythromycin effluxing strain by 32 and 16 times, respectively. Compound **11a** was tested against the SA-1199B strain of *S. aureus* that expresses the multidrug NorA efflux pump and found to inhibit EtBr efflux with an IC_50_ of 50 μM (Oluwatuyi et al., 2004). *J. elliptica* from the Euphorbiaceae family, distributed in North and the West of Brazil has shown several medicinal properties, and is used in treatment of several diseases. The antibacterial and resistance-modifying properties of 2,6-dimethyl-4-phenyl-pyridine- 3,5-dicarboxylic acid diethyl ester (**12**), derived from the rhizome of this plant, were investigated against *S. aureus* strains with NorA and MsrA efflux pumps. The findings revealed that the chemical inhibited the NorA efflux pump and enhanced activities of NorA substrates such as acriflavine, EtBr, and antibiotics like ciprofloxacin and norfloxacin (Marquez et al., 2005). Another compound kaempferol-3-*O-L*-(2,4-bis-*E-p*-coumaroyl) rhamnoside (**13**) isolated from *Persea lingue*, a traditional Chilean medicine, has inhibited the EtBr efflux at IC_50_ of 2 μM. Also, at concentration of 1.56 mg/L, compound has augmented the ciprofloxacin activity by eightfold in the strain over-expressing NorA pump and had no effect on ciprofloxacin activity in *norA* deleted mutant (Holler et al., 2012). Further no acute toxicity was detected in *in vivo* study (Ibrahim et al., 2009). Another plant *Wrightia tinctoria* (Apocynaceae family) native to India has been used to treat a variety of ailments. The EPI activity of Indirubin, one of the primary components in *W. tinctoria*, was examined against *S. aureus*. Indirubin enhanced the activity of ciprofloxacin synergistically with FICI (Fractional Inhibitory Concentration Index) of 0.45 and thus inhibited the Nor A efflux pump (Ponnusamy et al., 2010). The ability of Capsaicin as EPI was assessed in *S. aureus* strain over-expressing the NorA pump. Combination of ciprofloxacin (2 mg/L) with capsaicin (25 mg/L) reduced CFU by >3 log_10_ in 8 h, but ciprofloxacin alone at a significantly greater dose of 8 mg/L had the same effect. Furthermore, the post-antibiotic impact of ciprofloxacin was enhanced by 1.1 h when combined with capsaicin at MIC concentration (Kalia et al., 2012). Another flowering plant, *Hypericum olympicum* L. cf. *uniflorum* were evaluated and a new acylphloroglucinol antibacterial (**14**) compound was isolated, named as olympicin A. The compound **14** had shown the ability to impede NorA efflux pump and increased enoxacin accumulation (Shiu et al., 2013). *Alkanna orientalis* (L.) Boiss (Boraginaceae), a medicinal plant usually employed for the treatment of digestive problems (Lev and Amar, 2000). A flavonoid sarothrin (**15)** (5,7,4′-trihydroxy-3,6,8-trimethoxyflavone), was isolated from *A. orientalis* and has retarded the growth of *Mycobacterium smegmatis* (ATCC 607) with MIC of 75 μM and *S. aureus* (NCTC 8325-4) with MIC > 800 μM. EtBr efflux caused by the NorA efflux pump of *S. aureus* was inhibited by sarothrin at a concentration of 100 μM (Bame et al., 2013). Various members of the genus *Ammannia* are used to cure a variety of ailments in Chinese and Indian herbal medicine (Upadhyay et al., 2013). As a result, the bio-enhancing potential of *Ammannia* multiflora extracts and compounds has

been identified, with the 4-hydroxytetralone (**16**) and a number of its semi-synthetic aryl and acyl derivatives showing efficacy against nalidixic acid sensitive as well as resistant bacterial strains of *E. coli* (Upadhyay et al., 2012). Further study to understand its mechanistic approach was carried out. Two compounds from the study, compound **16** and its derivative **16a**, which has not shown its own antibacterial activity but when used along with tetracycline reduced the MIC of the tested antibiotic. Both these compounds inhibit the ATP dependent efflux pumps as the expression level of efflux pump gene, *yojI*, was down regulated, when used alone or in combination with tetracycline. Significant binding affinity of substances with YojI was reported in *in silico* docking studies, confirming these findings (Dwivedi et al., 2014). Figure 2B depicts the chemical structure of the natural EPIs **9–16a**.

In another study, the extract of milk thistle seed, Silybin (**17**), a flavonolignan component has shown the efficiency to inhibit the efflux mechanism of methicillin-resistant *S. aureus* (MRSA), MRSA41577 (Wang et al., 2018). The essential oils, dichloromethane extract (DCME), petroleum ether extracts, six compounds and two semi-synthetic derivatives of *Pilgerodendron uviferum* were tested as well for their ability to inhibit NorA efflux pumps in *S. aureus* strains namely, K2378, overexpressing *norA* gene (*norA*++). Out of all the compounds tested, only DCME and the compounds 15-copaenol (**18a**) and *epi*-cubenol (**18b**) inhibited EtBr efflux. All the combinations between tested antibiotics and *epi*-cubenol, DCME, and 15-copaenol, had exhibited synergism. Also, no toxicity was detected in HeLa cell line at the tested **EPIs** concentrations (Espinoza et al., 2019). Estragole (1-methoxy-allyl-benzene or 4-methoxyalyl-benzene), a monoterpene, has been reported to regulate the activity of norfloxacin and EtBr against *S. aureus* strains *in vivo* with no clinical symptoms of toxicity (da Costa et al., 2021). Other monoterpenes have been reported as well to have potentiating effects, such as thymol, that reduced the MIC of tetracycline, and eugenol, that reduced the MIC of norfloxacin against *S. aureus* (Sousa Silveira et al., 2020; Muniz et al., 2021). Pomegranate (*Punica granatum*) has also been shown to have antibacterial properties in traditional medicines for throat, diarrhoea and gum infections (Anesini and Perez, 1993). The synergistic interaction of *P. granatum* methanolic extract (PGME) with antibiotics was investigated on 30 MRSA and MSSA clinical isolates, and the findings revealed that PGME combined with chloramphenicol, ampicillin, gentamicin, oxacillin, and tetracycline, had a synergistic effect of 38–73%. PGME also increased the time it took for ampicillin to have a post-antibiotic effect (PAE) from 3 to 7 h and showed the ability to either inhibit or augment the drug’s inflow. However, in-depth mechanistic study is lacking (Braga et al., 2005).

Apart from the NorA efflux pump, the potential of natural EPI to inhibit other MDR efflux pumps has also been determined in few studies. EmrD-3, the member of MFS transporters family, display resistance for range of antimicrobial drugs in *V. cholerae*, including chloramphenicol, linezolid, erythromycin, minocycline, rifampin, rhodamine 6G, trimethoprim, and tetraphenyl phosphonium chloride (Smith et al., 2009). The antibacterial activities of garlic, *Allium sativum* extract and main bioactive component, allyl sulfide, was tested and its inhibitory activity for EmrD-3 efflux was also determined. Both were discovered to constrain efflux of EtBr in bacterial cells expressing EmrD-3, as well as lowered the MICs of a variety of antimicrobials, including fourfold decrease in linezolid, chloramphenicol, and erythromycin MICs, a 16-fold drop in MIC of tetracycline but kanamycin MIC increased by 33-fold. The more pronounced inhibitory effects of the extract and allyl sulfide have been seen on the toxigenic O1 *V. cholerae* strain N16961 (harboring EmrD-3) in comparison to non-toxigenic non-O1 strain PS15 (lacks EmrD-3) (Bruns et al., 2017). Cumin (*Cuminum cyminum L*.), a member of Apiaceae family, was tested as well for its capacity to block the LmrS (lincomycin multidrug resistance protein of *S. aureus*) pump. Cumin suppressed the development of *E. coli* KAM32 cells deficient in LmrS at a concentration of 25 mg/ml, but it’s methanolic extract, cuminaldehyde, and seed oil obstruct the progress of cells harboring LmrS at the MICs of 5, 12.5 μg/ml, and 25 mg/ml, respectively, implying that these compounds may dissolve the PMF (proton motive force) given rise by respiration. In *E. coli* KAM32 cells lacking as well as containing LmrS and ethidium efflux, a dose-dependent response to cumin treatment was detected (Floyd et al., 2010; Kakarla et al., 2017). Another study has assessed the library of 200 natural compounds displaying safe oral use, against multi-drug resistant *E. coli* isolate NCTC 13400. Among all, only four compounds named as ellagic acid, cepharanthine, propyl gallate and cinchonidine lowered the MICs of tetracycline, tobramycin and chloramphenicol by fourfold, and when combined lowered MIC up to eightfold. Only Cepharanthine has reduced the efflux of Nile red markers from cells. However, cepharanthine toxicity was identified in cultured HEK-293 mammalian cell-line models, while the other hit compounds showed no toxicity at the measured active doses (Jenic et al., 2020). The virtual screening from the library of 50 phytochemicals has identified Plumbagin, nordihydroguaretic (NDGA) (**19**) acid as a moderate EPI (Ohene-Agyei et al., 2014). Further analogs of NDGA were designed, synthesized and evaluated against *E. coli* BW25113 as AcrB inhibitors. Antibiotic resistance in *E. coli* and other Enterobacteriaceae is mostly due to the AcrAB-TolC efflux pump. Three analogs **20a, 20b,** and **20c** have shown potential as AcrB inhibitors, displaying increased potency as compared to NDGA. The extensively active analog **21c**, has been reported to improve the activity of four antibacterial classes: levofloxacin, chloramphenicol, erythromycin, and tetraphenylphosphonium (Wang et al., 2020). The chemical structure of the natural EPIs **17a-21** is shown in Figure 2C.

### Inhibitors of Modifying Enzymes

Resistance to antibiotics occurs through a variety of molecular mechanisms and enzymes play a vital role in all of these processes. For example, the most prevalent and admissible antibiotic resistance enzymes known are hydrolases that chops the β-lactam ring of antibiotics and makes them incompetent. Drug modification enzymes such as acetyltransferases, kinases and nucleotidyl transferases are also frequent (Schaenzer and Wright, 2020). Few natural inhibitors of enzymes have been discussed below:

Tea (*Camellia sinensis*), consumed by thousands of people worldwide has shown antimicrobial activity, especially its component epigallocatechin gallate (EGCg - compound **22**) (Toda et al., 1991; Ikigai et al., 1993). EGCg when combined at a concentration of less than 25 μg/ml with β-lactams, including benzylpenicillin, methicillin, oxacillin, ampicillin, and cephalexin has reversed the resistance of MRSA and the FIC indices observed varied from 0.126 to 0.625. Even in MSSA, EGCg has induced super susceptibility to β-lactams. EGCg with DL-cycloserine, the inhibitor of cell wall synthesis, has shown synergistic effect but PBP2* mRNA expression or production was not suppressed. Thus, the possible mechanism could be interference with the cell wall integrity (Zhao et al., 2001). Similarly, when EGCg was coupled with ampicillin/sulbactam, the susceptibility breakpoint against 28 clinical isolates of MRSA was reduced to 4 mg/L (Hu et al., 2001). At half the dose and below the MIC of 100 μg/ml, EGCg was also able to undo tetracycline resistance in Staphylococcal isolates harboring the Tet(K) efflux pump. It not only improved the MIC of tetracycline against resistance isolates but also against susceptible Staphylococcal isolates. Increased tetracycline accumulation inside bacterial cells was observed after Tet(K) pump function was inhibited (Sudano Roccaro et al., 2004).

Corilagin from *Arctostaphylos uva-ursi* and tellimagrandin I from *Rosa canina L.* have similar properties. In MRSA, (rose red) has shown resistance modifying activity for β-lactams. Since penicillin binding protein 2′ (2a) [PBP2′(PBP2a)], encoded by the *mecA* gene is linked to β-lactams resistance in MRSA. So, the effect of corilagin and tellimagrandin I on PBP2 was studied and the results suggested that these two inhibit the action of PBP2′(PBP2a) and the making of PBP2′(PBP2a) marginally. Further study indicated that in the presence of these two, PBP2′ (PBP2a) entirely lost the capability to bind penicillin and to PBP2 and PBP3 also but to a smaller magnitude. The compounds might have an effect on added aspects also involved in resistance of methicillin in MRSA, but the study regarding the same is lacking. Inactivation of PBPs, on the other hand, would restore β-lactams antibacterial action against MRSA (Shiota et al., 2004). Similar activity was also observed for baicalin (7-glucuronide of baicalein **23d**), a flavonoid obtained from Chinese herb *Scutellaria amoena* and other flavonoid such as apigenin (**23c**), luteolin (**23e**), or galangin (**23b**). The galangin (**23b**) made all of the strains of MRSA susceptible to amoxicillin and that their MICs were lowered from >2–250 μg/ml range to 0.25–2 μg/ml range. Galangin lowered the thickness of cell walls in comparison to control cells and likewise appeared to delay cell division. The resistance reversal activity also involves inhibition of β-lactamase activity. These flavonoids, alike other lactamase inhibitors, don’t contain a β-lactam ring and hence can reverse resistance to β-lactams by the use of possible several mechanisms (Eumkeb and Richards, 2005).

Further, a study has evaluated the potential of nineteen Jordanian plant extracts as modifying enzyme inhibitors. In folk medicine, the plants utilized in this study are often used to treat GI tract illnesses, skin diseases, and respiratory difficulties. The plant methanol extracts increased the diminishing effects of neomycin, chloramphenicol, doxycycline, nalidixic acid, and cephalexin against the standard as well as resistant strains of *E. coli.* The plant extracts with enhanced activity against resistant strains were *Gundelia tournefortii L*. and *Pimpinella anisum L.* (Darwish and Aburjai, 2010). Similarly, alkaloids obtained either from *Sophora flavescens Aiton* (Leguminosae) or *Sophora alopecuraides L.* have shown the antibacterial activities (Kuroyanagi et al., 1999; Chen et al., 2005; Cha et al., 2009). The investigation of reversal effects of *Sophora alopecuraides L.* (TASA) on the resistance of extended spectrum β-lactamases (ESBLs) producing *E. coli* has been studied. The MICs of TASA alone against the ESBLs producing strains was 12.5 mg/mL but the combination of its SIC with cefotaxime (CTX) and ceftazidime (CAZ) has shown synergistic effect. When isolates were exposed to lesser dosages of TASA, they were shown to be 8–16 times more susceptible to CTX and CAZ. Furthermore, enzymatic testing revealed that TASA induced reversal resistance to CTX and CAZ in these isolates in part by reducing the activation of the ESBLs. Molecular analysis also verified that no mutation in these isolates was detected in SHV-type beta-lactamase encoding ESBL gene, following exposure of TASA (Zhou et al., 2013). Another medicinal plant, *A. wilkesiana* commonly used for treating malaria, gastrointestinal problems, bacterial, and fungal infections (Alade and Irobi, 1993), managing cardiovascular disease (Omage et al., 2018), anti-inflammatory and analgesic effects (Olukunle et al., 2014) and sometimes cancers also (Lim et al., 2011) have shown the ability to reverse ampicillin resistance in MRSA. The antibacterial fraction 9EA-FC-B acquired from *A. wilkesiana* ethyl acetate crude extract showed synergistic effect with ampicillin in overcoming MRSA resistance by reducing PBP2a synthesis. The MIC of ampicillin was reduced by 32-fold, suggesting that 9EA-FC-B increased the sensitivity of MRSA against ampicillin. 9EA-FC-B either inactivated the PBP2a directly or inhibited the production of PBP2a since there was no expression of PBP2a in treated MRSA cultures (Santiago et al., 2014).

A group of scientists have also studied the effect of meropenem with almost 500 natural products to screen inhibitors of NDM-1 metallo-β-lactamases (MBLs) as this is mainly responsible for appearance of carbapenem-resistant gram-negative pathogens. The screening was done against the test strain, *E. coli* BW25113Δ*bamB*Δ*tolC*. Deletion of BamB and TolC increases the sensitivity of the screening as BamB is required for the assembly of outer membrane porin and its disruption increases the permeability for small molecules and TolC is the part of tripartite small molecule efflux systems, AcrA-AcrB-TolC that functions to remove tiny molecules from cell. Aspergillomarasmine A (AMA – compound **24**), fungus-based product, was established to be a strong inhibitor of enzyme NDM-1 and VIM-2, an additional MBL with clinical significance. Meropenem activity was also totally recovered in the presence of AMA against Enterobacteriaceae and *Pseudomonas spp.* as well as *Acinetobacter* spp. having alleles of the NDM or VIM types. In mice fed with *K. pneumoniae* that expresses NDM-1, the same effect was observed, showing that the combination of AMA with carbapenem antibiotics had therapeutic potential (King et al., 2014).

A potentiator of aminoglycoside antibiotics that inhibit enzyme modifying agents or targets has been the subject of a few studies. Recently, Yarlagadda et al. (2020) has screened the library of actinomycetes extracts in search of a compound that could restore the gentamicin activity against the gentamicin-resistant MRSA harboring bifunctional aminoglycoside-modifying enzymes (AME), AAC(6′)-Ie-APH(2″)-Ia. AME functions by phosphorylating and acetylating the antibiotic molecule and making it ineffective. Of all the compounds screened, one compound venturicidin A (VentA – compound **25**), a *Streptomyces*-derived molecule, has shown significant gentamicin enhancing activity. This molecule was discovered in 1961 and has shown antifungal activity by inhibiting ATP synthase (Rhodes et al., 1961; Perlin et al., 1985). MRSA, vancomycin-resistant enterococci (VRE), *K. pneumoniae*, *P. aeruginosa, E. coli*, and *A. baumannii* were tested for aminoglycoside-potentiation efficacy of VentA against aminoglycoside-resistant clinical isolates. VentA alone didn’t show its own antibacterial activity but decreased the MIC of gentamicin by 8–16-fold, when used in combination. The combination displays bactericidal activity and also rapidly eradicates MRSA. The mechanism of VentA action determined was concentration-dependent reduction in cellular ATP levels by 10-fold in MRSA, compared to untreated controls. It potentiates aminoglycosides activity by increasing the intracellular accumulation of antibiotics. Although VentA displayed toxicity *in vitro* toward HEK cells but in the mouse model, there was high tolerability when administered intraperitoneally (Yarlagadda et al., 2020). Recently, *n*-butanol has also been reported to potentiate the activity of aminoglycoside antibiotics, by rapidly enhancing the bacterial uptake of the antibiotic (Lv et al., 2022).

### Membrane Permeabilizer

The effective penetration of an antibiotic across the cell membrane potentiates its action. However, the decrease in cell membrane permeability being one of the intrinsic modes of resistance can be overcome by using membrane permeabilizers in conjunction with the antibiotics. The membrane permeabilizers are cationic and amphiphilic in nature that destabilizes the surface layer of the outer membrane by interacting with the polyanionic lipopolysaccharides or with outer layer cations, respectively. This results in enhanced permeability of the outer membrane ultimately assisting the drug uptake. Some of the examples of outer membrane permeabilizers are polymyxins, including colistin, aminoglycosides, cationic cholic acid derivatives, polyamines, cationic peptides etc. (Kwon and Lu, 2006). It has been recently reported that a commonly used EPI, phenylalanine-arginine β-naphthylamide (PaβN) in *P. aeruginosa* also has the ability to permeabilize bacterial membranes (González-Bello, 2017). Polymyxins including polymyxin B and polymyxin E (colistin) interact electrostatically with the bacterial outer membrane to displace Mg^2+^ and Ca^2+^ ions from their binding sites thus disrupting membrane integrity, causing cell damage and also enabling the influx of molecules like antibiotics (Landman et al., 2008). A combination of azithromycin with colistin was found to be effective against the MDR-isolates of *K. pneumoniae*, *P. aeruginosa*, and *A. baumannii* (Lin et al., 2015). Further, few of the recent synthetic chemical structures that potentiate antibiotics against Gram-negative bacteria by perturbing the OM have been reviewed extensively (Klobucar and Brown, 2022).

Various plant products that do not possess antibacterial activity, but act synergistic with the antibiotics by disrupting the outer membrane barriers seems promising. A promising study (Farrag et al., 2019) have shown gallic acid and thymol as potent permeabilizers against GNB, wherein these plant products were observed to potentiate the activity of different antibiotics such as azithromycin, erythromycin, nitrofurantoin, novobiocin, sulfamethoxazole, and trimethoprim. The combination of antibiotics and the permeabilizers decreased the MIC value and increased the overall susceptibility against the antibiotics tested (Farrag et al., 2019). The biosynthetic molecules such as terpenes and terpenoids, as well as other aliphatic and aromatic constituents with low molecular weight, found in essential oils (EOs) have shown to increase the penetration of antibiotics by permeabilizing the cell membrane, and thus have a potential to act as antibiotic adjuvants (Burt, 2004). The potential Citrus limon (EOCL) and Cinnamomum zeylanicum (EOCZ) essential oils as resistance altering compound has been investigated in a multidrug-resistant strain of *Acinetobacter* spp. The EOCL and EOCZ addition in growth medium at sub-inhibitory concentration leads to MIC reduction for meropenem, imipenem and amikacin. Both essential oils had a synergistic impact with amikacin, and additive effect with gentamicin (Guerra et al., 2012).

The effects of extracts/hydrolates from the number of traditional medicines on microbial pathogens have been studied. Hydrolates are made by steam distilling medicinal and fragrant plants and have been used in traditional medicine for therapy. The methanol root extract of *Nauclea pobeguinii*, methanol bark extract of *Albizia lebbeck*, seed hydrolate of *Aframomum sulcatum*, methanol bark extract of *Baillonella toxisperma* were evaluated for their potential to reverse antibiotic resistance against four antibiotics namely ceftriaxone, amoxicillin, ampicillin, and norfloxacin. Most of the extracts and hydrolates tested work in tandem with at least one antibiotic against at least one of the test species (Njimoh et al., 2015). Recently Mattingly et al. (2020), have screened the NCI Natural Products Set IV library in order to identify compounds showing antibiotic adjuvant activity against MRSA, *A. baumannii*, and *K. pneumoniae.* Adjuvant activity was screened using four classes of antibiotics- aminoglycosides, β-lactams, polymyxins, and macrolides. A total of 419 compounds were screened, and 64 of them were shown to be capable of potentiating the action of at least one of the antibiotics examined by reducing the MIC by four times. The highest compounds with antibiotic adjuvant activity have been identified with colistin against resistant strains of *A. baumannii* and *K. pneumoniae.* The MIC of colistin has been decreased by up to 1024-fold using nine different compounds and three compounds clorobiocin (**26)**, prodigiosin (**27**), and novobiocin (**28**) were the most potential ones. Colistin plus prodigiosin resulted in a massive upsurge in ROS levels (up to 1656-fold), which was not found in any other combination examined. But the exact mechanism by which natural compounds potentiate the activity of antibiotics has not been studied (Mattingly et al., 2020). Proanthocyanidins (cPAC) derived from American cranberry fruit (Vaccinium macrocarpon L.) have also been demonstrated to overcome ESBL-, MBL-, and PBP2a-mediated β-lactam resistance in clinical isolates of *Enterobacteriaceae* as well as ESBL- and MBL-producing *E. coli*. The cPAC has demonstrated activity for a variety of β-lactamases, including the CTX-M3 ESBL and the IMP-1 MBL. *In vivo* potency, on the other hand, has yet to be determined (Gallique et al., 2021). By enhancing outer membrane permeability and inhibiting efflux pumps, cPAC has been shown to augment the effectiveness of a variety of antibiotics against the opportunistic pathogens such as *E. coli, P. mirabilis*, and *P. aeruginosa* (Maisuria et al., 2019). The chemical structure of resistance enzyme inhibitor from **22-28** is shown in Figure 2D.

## Challenges and Limitations

Antibiotic potentiators still are in the early stage of development and face several challenges before being put into clinical applications. The first is the difficulty of medication formulation involving the combination of two pharmaceuticals and the requirement that the combined formulations have equivalent pharmacokinetic and pharmacodynamic properties. The effectiveness of β-lactam inhibitors is due to a thorough understanding of β-lactamase enzymes and their structural similarities to β-lactam antibiotics. The absence of other antibiotic adjuvants in clinical trials demonstrates a lack of such information and qualities (González-Bello, 2017). To mitigate the issue, methods such as covalent modification of antibiotics and the use of linkers to introduce potentiating compounds can be adopted.

Another downside of antibiotic potentiators is the risk for toxicity. Because of the toxicity involved with many of the compounds, some of the antibiotic potentiators with *in vitro* activity never make it to clinical trials. Furthermore, drug-drug interactions can have negative consequences. Another problem is ensuring optimal spatial and temporal dispersion of antibiotic-potentiator combinations at the target site. Delayed delivery of any of the molecules in the combination, or dissociation of the combination before it reaches the target location, will undoubtedly reduce the efficacy of this innovative strategy (Melander and Melander, 2017).

The occurrence of chromosomal or acquired resistance against potentiating molecules is a further concern to this strategy. Resistance to β-lactamase inhibitors has already been reported. The resistance against amoxicillin-clavulanic acid arises due to the overproduction of the sensitive β-lactamase, e.g., TEM-1 or SHV-1, resulting in lower susceptibility to clavulanic acid and other β-lactamase inhibitors (Stapleton et al., 1995). The other way to resistance could be due to the production of occasional “inhibitor-resistant” β-lactamases (Bush, 2010).

The research on the EPIs have been going through more than 20 years, however, they have been confined to laboratory use only (Verma et al., 2021). The main challenge for the plant-derived EPIs is their large size and complex structure. On the other hand the synthetic EPIs have toxicity, low solubility and poor cell permeability issues. Studies ensuring the specificity of potentiators are also required to minimize cytotoxicity in clinical applications. Furthermore, potentiator chemicals confront difficulties in clinical trials. The regulatory approval for Phase 2 clinical studies becomes challenging if the potentiating permeabilizer and companion antibiotic have no direct antibacterial activity (Vaara, 2019). These challenges and limitations make the antibiotic potentiators an unattractive investment for the pharmaceutical companies. However, the looming threat of AMR makes the discovery of antibiotic potentiators an urgent need. The antibiotic potentiators will surely help in the reversal of AMR and regenerate the antibiotics that have been discarded due to the emergence of resistance against them.

## Discussion

Antimicrobial resistance is a growing concern around the world, with the World Health Organisation (WHO) stating that the majority of Gram-negative bacteria are untreatable, imposing the progress of newer antimicrobials (Willyard, 2017). To tackle the issue, antibiotic resistance reversal chemicals are being developed and used as a complementary and alternative strategy to combat the problem. The review article has compiled a list of huge developments in the hunt for resistance-reversing compounds derived from natural and synthetic resources, as well as classified them according to their mode of action.

Much of the natural compounds with resistance reversal activity are defined for efflux-pumps and for β-lactamases. Inhibiting the efflux pumps will rejuvenate the use of antibiotics, which has developed resistance due to decreased effective concentration in cells. Huge success has been made for the development of β-lactamase inhibitors. Clavulanic acid is the one that is currently being used clinically since it inhibits the activity of several serine β-lactamases. However, Zn-dependent metallo β-lactamases from class B, AmpCs serine β-lactamases from class C, and OXAs serine β-lactamases from class D are not inhibited by Clavulanic acid and thus limits its potential and efficacy (Drawz and Bonomo, 2010).

Significant research for developing plasmid-curing compounds of natural origin has also been performed, but none of these have made it to the clinic yet. Methods for significantly and safely eliminating plasmids have the prospect of diminishing the severity of drug-resistant infections. By speeding up the hunt for resistance reversal inhibitors that target other processes as well, there is sufficient opportunity to increase the use of current antibiotics even more. The mechanism of conjugal plasmid transfer inhibition by natural resources is less explored. Conjugative plasmids are the primary vehicle for the transmission of AMR genes. So, their inhibition will diminish the transfer efficiency of AMR genes amongst bacterial communities (Cabezón et al., 2017).

Currently, only a few studies have looked at the chemicals’ potential *in vivo*. Further *in vivo* investigations will be critical in developing strategies to sensitize infectious bacteria to a wide spectrum of antibiotics, as well as providing important information about the compounds’ efficiency in a living creature. Future research should concentrate on elucidating and comprehending the chemicals’ deep mechanistic approach. Plant extracts with potential activity can also be used for compound discovery, characterization, and modification. However, the developments of resistance in continually evolving bacteria, as well as the influence of these techniques on bacterial community structure, are long-term concerns for these resistance reversal agents. Supplementing with evolution-proof drugs is incredibly difficult, but we can certainly slow down the process by looking for resistance-reversing compounds in nature’s library. Future research in this area is expected to continue, owing to the necessity to restore susceptibility to current antibiotics in order to cure resistance illnesses.

## Conclusion

Widespread resistance to available antibiotics in clinically important pathogenic bacteria is currently a global challenge due to an ever-increasing number of resistant strains to multiple antibiotics. To address this problem, the use of “antibiotic adjuvants/potentiators” in combination with antibiotics is now being exploited. Nature and its natural products along with various synthetic compounds have come to our rescue. Nature has been a storehouse of indispensable materials that sustain life on this planet. Once appraised as the magic bullets, antibiotics were discovered from various natural resources. However, the discovery of the antibiotics couldn’t keep pace with the subsequent emergence of resistance. Various plant-derived as well as synthetic compounds have shown to enhance the action potential of the antibiotics used and they act against the resistance mechanism employed by the pathogen. Most of the compounds have been tested under *in vitro* conditions; however, there is paucity of conclusive data regarding their spectrum of activity, mode of action, toxicity and *in vivo* efficacy. Future studies will surely pave the way ahead to reverse antibiotic resistance and rejuvenate the arsenal of antibiotics in hand.

## Author Contributions

BD, MC, and JV designed the research. MC, JV, RG, and BD wrote the review. BD reviewed and edited the manuscript. All authors contributed to the article and approved the submitted version.

## Conflict of Interest

The authors declare that the research was conducted in the absence of any commercial or financial relationships that could be construed as a potential conflict of interest.

## Publisher’s Note

All claims expressed in this article are solely those of the authors and do not necessarily represent those of their affiliated organizations, or those of the publisher, the editors and the reviewers. Any product that may be evaluated in this article, or claim that may be made by its manufacturer, is not guaranteed or endorsed by the publisher.

## References

[B1] Alade P. I. Irobi O. N. (1993). Antimicrobial activities of crude leaf extracts of Acalypha wilkesiana. *J.Ethnopharmacol.* 39 171–174. 10.1016/0378-8741(93)90033-2 8258974

[B2] Amábile Cuevas C. F. (1988). Loss of penicillinase plasmids of Staphylococcus aureus after treatment with L-ascorbic acid. *Mutation Res.* 207 107–109. 10.1016/0165-7992(88)90072-3 3258647

[B3] Amábile-Cuevas C. F. Piña-Zentella R. M. Wah-Laborde M. E. (1991). Decreased reistance to antibiotics and plasmid loss in plasmid-carrying strains of Staphylocccus aureus treated with ascorbic acid. *Mutation Res. Lett.* 119–125. 10.1016/0165-7992(91)90128-q 1944394

[B4] Anderson E. S. Threlfall E. J. Carr J. M. Savoy L. G. (1973). Bacteriophage restriction in *Salmonella* typhimurium by R factors and transfer factors. *J. Hygiene* 71 619–631. 10.1017/s0022172400046611 4584174 PMC2130588

[B5] Anesini C. Perez C. (1993). Screening of plants used in Argentine folk medicine for antimicrobial activity. *J. Ethnopharmacol.* 39 119–128. 10.1016/0378-8741(93)90027-3 8412245

[B6] Arzanlou M. Chai W. C. Venter H. (2017). Intrinsic, adaptive and acquired antimicrobial resistance in Gram-negative bacteria. *Essays. Biochem.* 61 49–59. 10.1042/EBC20160063 28258229

[B7] Astolfi A. Felicetti T. Iraci N. Manfroni G. Massari S. Pietrella D. (2017). Pharmacophore-Based Repositioning of Approved Drugs as NovelStaphylococcus aureusNorA Efflux Pump Inhibitors. *J. Med. Chem.* 60 1598–1604. 10.1021/acs.jmedchem.6b01439 28117588

[B8] Bakken J. S. Borody T. Brandt L. J. Brill J. V. Demarco D. C. Franzos M. A. (2011). Treating Clostridium difficile infection with fecal microbiota transplantation. *Clin. Gastroenterol. Hepatol.* 9 1044–1049. 21871249 10.1016/j.cgh.2011.08.014 PMC3223289

[B9] Bame J. R. Graf T. N. Junio H. A. Bussey R. O. III Jarmusch S. A. El-Elimat T. (2013). Sarothrin from Alkanna orientalis is an antimicrobial agent and efflux pump inhibitor. *Planta Med.* 79 327–329. 10.1055/s-0032-1328259 23468310 PMC4527991

[B10] Bennett P. M. (1999). Integrons and gene cassettes: a genetic construction kit for bacteria. *J. Antimicrobial. Chemother.* 43 1–4. 10381094

[B11] Bennett P. M. (2008). Plasmid encoded antibiotic resistance: acquisition and transfer of antibiotic resistance genes in bacteria. *Br. J. Pharmacol.* 153 S347–S357. 10.1038/sj.bjp.0707607 18193080 PMC2268074

[B12] Bettiol E. Wetherington J. D. Schmitt N. Harbarth S. Combacte Consortium. (2015). Challenges and solutions for clinical development of new antibacterial agents: results of a survey among pharmaceutical industry professionals. *Antimicrob. Agents Chemother.* 59 3695–3699. 10.1128/AAC.00638-15 25918147 PMC4468719

[B13] Bhardwaj A. K. Mohanty P. (2012). Bacterial efflux pumps involved in multidrug resistance and their inhibitors: rejuvinating the antimicrobial chemotherapy’. *Recent Pat. Antiinfect. Drug Discov.* 7 73–89. 10.2174/157489112799829710 22353004

[B14] Blagden C. O. (1937). A Dlctionary of the Economic Products of the Malay Peninsula. By J. H. Burkill, with contributions by William Birtwistle, F. W. Foxworthy, J. B. Scrivenor, and J. G. Watson. 2 vols. 9 × 6. pp. xi 2402. London: Published on behalf of the Governments of the Straits Settlements and Federated Malay States by the Crown Agents for the Colonies, 1935. 30s’. *J. R. Asiatic Soc. Great Br. Ireland* 69 134–135. 10.1017/s0035869x00096325

[B15] Blair J. M. A. Richmond G. E. Piddock L. J. V. (2014). Multidrug efflux pumps in Gram-negative bacteria and their role in antibiotic resistance. *Future Microbiol.* 9 1165–1177. 25405886 10.2217/fmb.14.66

[B16] Blanco M. G. Hardisson C. Salas J. A. (1984). Resistance in inhibitors of RNA polymerase in actinomycetes which produce them. *J. Gen. Microbiol.* 130 2883–2891. 10.1099/00221287-130-11-2883 6084703

[B17] Braga L. C. Leite A. A. M. Xavier K. G. S. Takahashi J. A. Bemquerer M. P. Chartone-Souza E. (2005). Synergic interaction between pomegranate extract and antibiotics against Staphylococcus aureus. *Can. J. Microbiol.* 51 541–547. 10.1139/w05-022 16175202

[B18] Brown A. G. (1986). Clavulanic acid, a novel beta-lactamase inhibitor–a case study in drug discovery and development. *Drug Design Delivery* 1 1–21. 3334541

[B19] Bruns M. M. Kakarla P. Floyd J. T. Mukherjee M. M. Ponce R. C. Garcia J. A. (2017). Modulation of the multidrug efflux pump EmrD-3 from *Vibrio cholerae* by Allium sativum extract and the bioactive agent allyl sulfide plus synergistic enhancement of antimicrobial susceptibility by A. sativum extract. *Arch. Microbiol.* 199 1103–1112. 10.1007/s00203-017-1378-x 28432381

[B20] Burrus V. Pavlovic G. Decaris B. Guédon G. (2002). Conjugative transposons: the tip of the iceberg. *Mol. Microbiol.* 46 601–610. 10.1046/j.1365-2958.2002.03191.x 12410819

[B21] Burt S. (2004). Essential oils: their antibacterial properties and potential applications in foods—a review. *Int. J. Food Microbiol.* 223–253. 10.1016/j.ijfoodmicro.2004.03.022 15246235

[B22] Bush K. (2010). Other β-lactam antibiotics. *Antibiotic Chemother.* 226–244. 10.1016/b978-0-7020-4064-1.00015-4

[B23] Cabezón E. de la Cruz F. Arechaga I. (2017). Conjugation Inhibitors and Their Potential Use to Prevent Dissemination of Antibiotic Resistance Genes in Bacteria. *Front. Microbiol.* 8:2329. 10.3389/fmicb.2017.02329 29255449 PMC5723004

[B24] Campo J. D. E. L. del Campo J. Amiot M.-J. Christophe N. G. U. Y. E. N.-T. H. E. (2000). Antimicrobial Effect of Rosemary Extracts. *J. Food Protect.* 63 1359–1368. 10.4315/0362-028x-63.10.1359 11041135

[B25] Carattoli A. (2009). Resistance plasmid families in *Enterobacteriaceae*. *Antimicrobial. Agents Chemother.* 53 2227–2238. 10.1128/AAC.01707-08 PMC2687249 19307361

[B26] Carattoli A. (2013). Plasmids and the spread of resistance. *Int. J. Med. Microbiol.* 303 298–304. 23499304 10.1016/j.ijmm.2013.02.001

[B27] Cha J.-D. Moon S.-E. Kim J.-Y. Jung E.-K. Lee Y.-S. (2009). Antibacterial activity of sophoraflavanone G isolated from the roots of Sophora flavescens against methicillin-resistant Staphylococcus aureus. *Phytother. Res.* 23 1326–1331. 10.1002/ptr.2540 19288534

[B28] Chandra H. Bishnoi P. Yadav A. Patni B. Mishra A. Nautiyal A. (2017). Antimicrobial Resistance and the Alternative Resources with Special Emphasis on Plant-Based Antimicrobials—A Review. *Plants* 6:16. 10.3390/plants6020016 PMC5489788 28394295

[B29] Chen L. Cheng X. Shi W. Lu Q. Go V. L. Heber D. (2005). Inhibition of Growth of Streptococcus mutans, Methicillin-Resistant Staphylococcus aureus, and Vancomycin-Resistant Enterococci by Kurarinone, a Bioactive Flavonoid Isolated from Sophora flavescens. *J. Clin. Microbiol.* 43 3574–3575. 10.1128/jcm.43.7.3574-3575.2005 16000511 PMC1169149

[B30] Chitemerere T. A. Mukanganyama S. (2014). Evaluation of cell membrane integrity as a potential antimicrobial target for plant products. *BMC Compl. Alternative Med.* 14:278. 10.1186/1472-6882-14-278 PMC4124163 25078023

[B31] Choi U. Lee C.-R. (2019). Distinct Roles of Outer Membrane Porins in Antibiotic Resistance and Membrane Integrity in *Escherichia coli*. *Front. Microbiol.* 10:953. 10.3389/fmicb.2019.00953 31114568 PMC6503746

[B32] Cock I. (2012). Research Letter Antimicrobial activity of Callistemon citrinus and Callistemon salignus methanolic extracts. *Pharmacognosy Commun.* 2 50–57. 10.5530/pc.2012.3.11

[B33] Costa S. S. Viveiros M. Amaral L. Couto I. (2013). Multidrug Efflux Pumps in Staphylococcus aureus: an Update. *Open Microbiol. J.* 7 59–71. 10.2174/1874285801307010059 23569469 PMC3617543

[B34] Cox G. Wright G. D. (2013). Intrinsic antibiotic resistance: mechanisms, origins, challenges and solutions. *Int. J. Med. Microbiol.* 303 287–292. 10.1016/j.ijmm.2013.02.009 23499305

[B35] da Costa R. H. S. Rocha J. E. de Freitas T. S. Pereira R. L. S. Junior F. N. P. de Oliveira M. R. C. (2021). Evaluation of antibacterial activity and reversal of the NorA and MepA efflux pump of estragole against Staphylococcus aureus bacteria. *Arch. Microbiol.* 203 3551–3555. 10.1007/s00203-021-02347-x 33942156

[B36] Darwish R. M. Aburjai T. A. (2010). Effect of ethnomedicinal plants used in folklore medicine in Jordan as antibiotic resistant inhibitors on *Escherichia coli*. *BMC Complementary Alternative Med.* 10:9. 10.1186/1472-6882-10-9 PMC2839964 20187978

[B37] Datta N. Hedges R. W. (1971). Compatibility groups among fi - R factors. *Nature* 234 222–223. 10.1038/234222a0 5002028

[B38] Drawz S. M. Bonomo R. A. (2010). Three decades of beta-lactamase inhibitors. *Clin. Microbiol. Rev.* 23 160–201. 10.1128/CMR.00037-09 20065329 PMC2806661

[B39] Dwivedi G. R. Upadhyay H. C. Yadav D. K. Singh V. Srivastava S. K. Khan F. (2014). 4-Hydroxy-α-tetralone and its derivative as drug resistance reversal agents in multi drug resistant *Escherichia coli*. *Chem. Biol. Drug Des.* 83 482–492. 10.1111/cbdd.12263 24267788

[B40] Espinoza J. Urzúa A. Sanhueza L. Walter M. Fincheira P. Muñoz P. (2019). Essential Oil, Extracts, and Sesquiterpenes Obtained From the Heartwood of Act as Potential Inhibitors of the NorA Multidrug Efflux Pump. *Front. Microbiol.* 10:337. 10.3389/fmicb.2019.00337 30863385 PMC6400098

[B41] Eumkeb G. Richards R. M. F. (2005). REVERSING BETA-LACTAM ANTIBIOTIC RESISTANCE WITH FLAVONOIDS IN GRAM-POSITIVE BACTERIA. *Acta Horticulturae* 678 171–178. 10.17660/actahortic.2005.678.24

[B42] Farrag H. A. Abdallah N. Shehata M. M. K. Awad E. M. (2019). Natural outer membrane permeabilizers boost antibiotic action against irradiated resistant bacteria. *J. Biomed. Sci.* 26:69. 10.1186/s12929-019-0561-6 31500622 PMC6732830

[B43] Floyd J. L. Smith K. P. Kumar S. H. Floyd J. T. Varela M. F. (2010). LmrS is a multidrug efflux pump of the major facilitator superfamily from Staphylococcus aureus. *Antimicrob. Agents Chemother.* 54 5406–5412. 10.1128/AAC.00580-10 20855745 PMC2981259

[B44] Foster T. J. Davis M. A. Roberts D. E. Takeshita K. Kleckner N. (1981). Genetic organization of transposon Tn10. *Cell* 23 201–213. 10.1016/0092-8674(81)90285-3 6260375

[B45] Gallique M. Wei K. Maisuria V. B. Okshevsky M. McKay G. Nguyen D. (2021). Cranberry-Derived Proanthocyanidins Potentiate β-Lactam Antibiotics against Resistant Bacteria. *Appl. Environ. Microbiol* 87 e00127–21. 10.1128/AEM.00127-21 33712420 PMC8117774

[B46] Getino M. Fernández-López R. Palencia-Gándara C. Campos-Gómez J. Sánchez-López J. M. Martínez M. (2016). Tanzawaic Acids, a Chemically Novel Set of Bacterial Conjugation Inhibitors. *PLoS One* 11:e0148098. 10.1371/journal.pone.0148098 26812051 PMC4727781

[B47] Gibbons S. Oluwatuyi M. Veitch N. C. Gray A. I. (2003). Bacterial resistance modifying agents from Lycopus europaeus. *Phytochemistry* 62 83–87. 10.1016/s0031-9422(02)00446-6 12475623

[B48] González-Bello C. (2017). Antibiotic adjuvants - A strategy to unlock bacterial resistance to antibiotics. *Bioorganic Med. Chem. Lett.* 27 4221–4228. 10.1016/j.bmcl.2017.08.027 28827113

[B49] Gorgani N. Ahlbrand S. Patterson A. Pourmand N. (2009). Detection of point mutations associated with antibiotic resistance in *Pseudomonas aeruginosa*. *Int. J. Antimicrob. Agents* 34 414–418. 10.1016/j.ijantimicag.2009.05.013 19656662 PMC2744841

[B50] Guerra F. Q. S. Mendes J. M. Sousa J. P. de, Morais-Braga M. F. B. Santos B. H. C. (2012). Increasing antibiotic activity against a multidrug-resistant Acinetobacter spp by essential oils of Citrus limon and Cinnamomum zeylanicum. *Nat. Prod. Res.* 26 2235–2238. 10.1080/14786419.2011.647019 22191514

[B51] Hansen J. L. Moore P. B. Steitz T. A. (2003). Structures of Five Antibiotics Bound at the Peptidyl Transferase Center of the Large Ribosomal Subunit. *J. Mol. Biol.* 1061–1075. 10.1016/s0022-2836(03)00668-5 12860128

[B52] He J.-M. Sun S.-C. Sun Z.-L. Chen J.-T. Mu Q. (2019). Isovalerylshikonin, a new resistance-modifying agent from Arnebia euchroma, supresses antimicrobial resistance of drug-resistant Staphylococcus aureus. *Int. J. Antimicrob. Agents* 53 70–73. 10.1016/j.ijantimicag.2018.08.021 30176356

[B53] Herelle F. d’ Smith G. H. (1922). *The Bacteriophage, Its Rôle In Immunity.* Wyoming:Creative Media Partners, LLC 10.5962/bhl.title.31194

[B54] Holler J. G. Christensen S. B. Slotved H.-C. Rasmussen H. B. Gúzman A. Olsen C.-E. (2012). Novel inhibitory activity of the Staphylococcus aureus NorA efflux pump by a kaempferol rhamnoside isolated from Persea lingue Nees. *J. Antimicrob. Chemother.* 67 1138–1144. 10.1093/jac/dks005 22311936

[B55] Hu Z. Q. Zhao W. H. Hara Y. Shimamura T. (2001). Epigallocatechin gallate synergy with ampicillin/sulbactam against 28 clinical isolates of methicillin-resistant Staphylococcus aureus. *J. Antimicrob. Chemother.* 48 361–364. 10.1093/jac/48.3.361 11533000

[B56] Hussein A. A. Rodríguez B. (2000). Isopimarane diterpenoids from Lycopus europaeus. *J. Nat. Products* 63 419–421. 10.1021/np990519c 10757737

[B57] Ibrahim M. A. Mansoor A. A. Gross A. Ashfaq M. K. Jacob M. Khan S. I. (2009). Methicillin-resistant Staphylococcus aureus (MRSA)-active metabolites from Platanus occidentalis (American Sycamore). *J. Nat. Prod.* 72 2141–2144. 10.1021/np900499q 19904995 PMC4883668

[B58] Ikigai H. Nakae T. Hara Y. Shimamura T. (1993). Bactericidal catechins damage the lipid bilayer. *Biochim. Biophys. Acta* 1147 132–136. 10.1016/0005-2736(93)90323-r 8466924

[B59] Jenic D. Waller H. Collins H. Erridge C. (2020). Reversal of Tetracycline Resistance by Cepharanthine, Cinchonidine, Ellagic Acid and Propyl Gallate in a Multi-drug Resistant *Escherichia coli*. *Natural Prod. Bioprospect.* 11 345–355. 10.1007/s13659-020-00280-y PMC8141080 33141306

[B60] Jenssen H. Hamill P. Hancock R. E. W. (2006). Peptide Antimicrobial Agents. *Clin. Microbiol. Rev.* 491–511. 10.1128/cmr.00056-05 16847082 PMC1539102

[B61] Jo A. Ahn J. (2016). Phenotypic and genotypic characterisation of multiple antibiotic-resistant Staphylococcus aureus exposed to subinhibitory levels of oxacillin and levofloxacin. *BMC Microbiol.* 16:170. 10.1186/s12866-016-0791-7 27473500 PMC4966875

[B62] Johnson C. M. Grossman A. D. (2015). Integrative and Conjugative Elements (ICEs): What They Do and How They Work. *Ann. Rev. Gene.* 49 577–601. 10.1146/annurev-genet-112414-055018 PMC5180612 26473380

[B63] Kakarla P. Floyd J. Mukherjee M. Devireddy A. R. Inupakutika M. A. Ranweera I. (2017). Inhibition of the multidrug efflux pump LmrS from Staphylococcus aureus by cumin spice Cuminum cyminum. *Arch. Microbiol.* 199 465–474. 10.1007/s00203-016-1314-5 27830269

[B64] Kalia N. P. Mahajan P. Mehra R. Nargotra A. Sharma J. P. Koul S. (2012). Capsaicin, a novel inhibitor of the NorA efflux pump, reduces the intracellular invasion of Staphylococcus aureus. *J. Antimicrob. Chemother.* 67 2401–2408. 10.1093/jac/dks232 22807321

[B65] King A. M. Reid-Yu S. A. Wang W. King D. T. De Pascale G. Strynadka N. C. (2014). Aspergillomarasmine A overcomes metallo-β-lactamase antibiotic resistance. *Nature* 510 503–506. 10.1038/nature13445 24965651 PMC4981499

[B66] Klobucar K. Brown E. D. (2022). New potentiators of ineffective antibiotics: Targeting the Gram-negative outer membrane to overcome intrinsic resistance. *Curr. Opin. Chem. Biol.* 66:102099. 10.1016/j.cbpa.2021.102099 34808425

[B67] Kuroyanagi M. Arakawa T. Hirayama Y. Hayashi T. (1999). Antibacterial and antiandrogen flavonoids from Sophora flavescens. *J. Nat. Prod.* 62 1595–1599. 10.1021/np990051d 10654410

[B68] Kwon D. H. Lu C.-D. (2006). Polyamines increase antibiotic susceptibility in *Pseudomonas aeruginosa*. *Antimicrobial. Agents Chemother.* 50 1623–1627. 10.1128/AAC.50.5.1623-1627.2006 16641427 PMC1472196

[B69] Lakhmi V. V. Padma S. Polasa H. (1987). Elimination of multidrug-resistant plasmid in bacteria by plumbagin, a compound derived from a plant. *Curr. Microbiol.* 159–161. 10.1007/bf01568396

[B70] Landman D. Georgescu C. Martin D. A. Quale J. (2008). Polymyxins revisited. *Clin. Microbiol. Rev.* 21 449–465. 10.1128/CMR.00006-08 18625681 PMC2493081

[B71] Latha C. Shriram V. D. Jahagirdar S. S. Dhakephalkar P. K. Rojatkar S. R. (2009). ‘Antiplasmid activity of 1’-acetoxychavicol acetate from Alpinia galanga against multi-drug resistant bacteria.’. *J. Ethnopharmacol.* 123 522–525. 10.1016/j.jep.2009.03.028 19501283

[B72] Laws M. Shaaban A. Rahman K. M. (2019). Antibiotic resistance breakers: current approaches and future directions. *FEMS Microbiol. Rev.* 43 490–516. 10.1093/femsre/fuz014 31150547 PMC6736374

[B73] Leclercq R. (2002). Mechanisms of resistance to macrolides and lincosamides: nature of the resistance elements and their clinical implications. *Clin. Infect. Dis.* 34 482–492. 10.1086/324626 11797175

[B74] Lee N. Yuen K.-Y. Kumana C. R. (2003). Clinical role of beta-lactam/beta-lactamase inhibitor combinations. *Drugs* 63 1511–1524. 12834367 10.2165/00003495-200363140-00006

[B75] Lev E. Amar Z. (2000). Ethnopharmacological survey of traditional drugs sold in Israel at the end of the 20th century. *J. Ethnopharmacol.* 72 191–205. 10.1016/s0378-8741(00)00230-0 10967472

[B76] Li H. Luo Y.-F. Williams B. J. Blackwell T. S. Xie C.-M. (2012). Structure and function of OprD protein in *Pseudomonas aeruginosa*: from antibiotic resistance to novel therapies. *Int. J. Med. Microbiol.* 302 63–68. 10.1016/j.ijmm.2011.10.001 22226846 PMC3831278

[B77] Li X.-Z. Plésiat P. Nikaido H. (2015). The challenge of efflux-mediated antibiotic resistance in Gram-negative bacteria. *Clin. Microbiol. Rev.* 28 337–418. 10.1128/CMR.00117-14 25788514 PMC4402952

[B78] Lim S. W. Ting K. N. Bradshaw T. D. Zeenathul N. A. Wiart C. Khoo T. J. (2011). Acalypha wilkesiana extracts induce apoptosis by causing single strand and double strand DNA breaks. *J. Ethnopharmacol.* 138 616–623. 10.1016/j.jep.2011.10.005 22008878

[B79] Lin L. Nonejuie P. Munguia J. Hollands A. Olson J. Dam Q. (2015). Azithromycin Synergizes with Cationic Antimicrobial Peptides to Exert Bactericidal and Therapeutic Activity Against Highly Multidrug-Resistant Gram-Negative Bacterial Pathogens. *EBioMedicine* 2 690–698. 10.1016/j.ebiom.2015.05.021 26288841 PMC4534682

[B80] Lomovskaya O. Bostian K. A. (2006). Practical applications and feasibility of efflux pump inhibitors in the clinic–a vision for applied use. *Biochem. Pharmacol.* 71 910–918. 10.1016/j.bcp.2005.12.008 16427026

[B81] Lopatkin A. J. Meredith H. R. Srimani J. K. Pfeiffer C. Durrett R. You L. (2017). Persistence and reversal of plasmid-mediated antibiotic resistance. *Nat. Commun.* 8:1689. 10.1038/s41467-017-01532-1 29162798 PMC5698434

[B82] Lv B. Bian M. Huang X. Sun F. Gao Y. Wang Y. (2022). -Butanol Potentiates Subinhibitory Aminoglycosides against Bacterial Persisters and Multidrug-Resistant MRSA by Rapidly Enhancing Antibiotic Uptake. *ACS Infect. Dis.* 8 373–386. 10.1021/acsinfecdis.1c00559 35100802

[B83] Maisuria V. B. Okshevsky M. Déziel E. Tufenkji N. (2019). Proanthocyanidin Interferes with Intrinsic Antibiotic Resistance Mechanisms of Gram-Negative Bacteria. *Adv. Sci.* 6:1802333. 10.1002/advs.201802333 31406662 PMC6685479

[B84] Marquez B. Neuville L. Moreau N. J. Genet J.-P. dos Santos A. F. Caño (2005). Multidrug resistance reversal agent from Jatropha elliptica. *Phytochemistry* 66 1804–1811. 10.1016/j.phytochem.2005.06.008 16051285

[B85] Martins M. Dastidar S. G. Fanning S. Kristiansen J. E. Molnar J. Pagès J.-M. (2008). Potential role of non-antibiotics (helper compounds) in the treatment of multidrug-resistant Gram-negative infections: mechanisms for their direct and indirect activities. *Int. J. Antimicrob. Agents* 31 198–208. 10.1016/j.ijantimicag.2007.10.025 18180147

[B86] Mattingly A. E. Cox K. E. Smith R. Melander R. J. Ernst R. K. Melander C. (2020). Screening an Established Natural Product Library Identifies Secondary Metabolites That Potentiate Conventional Antibiotics. *ACS Infect Dis.* 6 2629–2640. 10.1021/acsinfecdis.0c00259 32810395 PMC8330956

[B87] McCusker M. P. Alves Ferreira D. Cooney D. Martins Alves B. Fanning S. Pagès J.-M. (2019). Modulation of antimicrobial resistance in clinical isolates of *Enterobacter* aerogenes: A strategy combining antibiotics and chemosensitisers. *J. Glob. Antimicrob. Resist.* 16 187–198. 30321623 10.1016/j.jgar.2018.10.009

[B88] Melander R. J. Melander C. (2017). The Challenge of Overcoming Antibiotic Resistance: An Adjuvant Approach? *ACS Infect. Dis.* 3 559–563. 10.1021/acsinfecdis.7b00071 28548487 PMC5798239

[B89] Meynell E. Meynell G. G. Datta N. (1968). Phylogenetic relationships of drug-resistance factors and other transmissible bacterial plasmids. *Bacteriol. Rev.* 32 55–83. 10.1128/br.32.1.55-83.1968 4869941 PMC378292

[B90] Morgan A. R. Cone R. L. Terry M. E. (1976). The mechanism of DNA strand breakage by vitamin C and superoxide and the protective roles of catalase and superoxide dismutase. *Nucleic Acids Res.* 3, 1139–1150. 10.1093/nar/3.5.1139 181730 PMC342976

[B91] Mozirandi W. Tagwireyi D. Mukanganyama S. (2019). Evaluation of antimicrobial activity of chondrillasterol isolated from Vernonia adoensis (Asteraceae). *BMC Complement. Alternative Med.* 19:249. 10.1186/s12906-019-2657-7 PMC6731578 31492140

[B92] Munita J. M. Arias C. A. (2016). Mechanisms of Antibiotic Resistance. *Microbiol. Spectrum* 4:10.1128/microbiolspec.VMBF-0016-2015. 10.1128/microbiolspec.VMBF-0016-2015 PMC4888801 27227291

[B93] Muniz D. F. Dos Santos Barbosa C. R. de Menezes I. R. A. de Sousa E. O. Pereira R. L. S. Júnior J. T. C. (2021). In vitro and in silico inhibitory effects of synthetic and natural eugenol derivatives against the NorA efflux pump in Staphylococcus aureus. *Food Chem.* 337:127776. 10.1016/j.foodchem.2020.127776 32777574

[B94] Nikaido H. (2003). Molecular basis of bacterial outer membrane permeability revisited’. *Microbiol. Mol. Biol. Rev.* 67 593–656. 10.1128/MMBR.67.4.593-656.2003 14665678 PMC309051

[B95] Njimoh D. L. Assob J. C. N. Mokake S. E. Nyhalah D. J. Yinda C. K. Sandjon B. (2015). Antimicrobial Activities of a Plethora of Medicinal Plant Extracts and Hydrolates against Human Pathogens and Their Potential to Reverse Antibiotic Resistance. *Int. J. Microbiol.* 2015:547156. 10.1155/2015/547156 26180528 PMC4477429

[B96] Novick R. P. Clowes R. C. Cohen S. N. Curtiss R. Datta N. Falkow S. (1976). Uniform nomenclature for bacterial plasmids: a proposal. *Bacteriol. Rev.* 40 168–189. 10.1128/br.40.1.168-189.1976 1267736 PMC413948

[B97] Ohene-Agyei T. Mowla R. Rahman T. Venter H. (2014). Phytochemicals increase the antibacterial activity of antibiotics by acting on a drug efflux pump. *Microbiologyopen* 3 885–896. 10.1002/mbo3.212 25224951 PMC4263512

[B98] Oliveira M. M. Santos H. S. Coutinho H. D. M. Bandeira P. N. da Silva P. T. Freitas T. S. (2020). Spectroscopic characterization and efflux pump modulation of a thiophene curcumin derivative. *J. Mol. Struct.* 1215:128291. 10.1016/j.molstruc.2020.128291

[B99] Olukunle J. O. Adenubi O. T. Biobaku K. T. Oyewusi J. A. Arowolo R. O. A. (2014). Anti-inflammatory and analgesic effects of methanol extract and fractions of Acalypha wilkesiana leaves. *Planta Med.* 80 181–4. 10.1055/s-0034-1395071 24285125

[B100] Oluwatuyi M. Kaatz G. W. Gibbons S. (2004). Antibacterial and resistance modifying activity of Rosmarinus officinalis. *Phytochemistry* 65 3249–3254. 10.1016/j.phytochem.2004.10.009 15561190

[B101] Omage K. Azeke M. A. Omage S. O. (2018). Evaluation of the efficacy of Acalypha wilkesiana leaves in managing cardiovascular disease risk factors in rabbits exposed to salt-loaded diets. *Clin. Phytosci.* 4:1. 10.1186/s40816-018-0060-4

[B102] Oyedemi B. O. Kotsia E. M. Stapleton P. D. Gibbons S. (2019). Capsaicin and gingerol analogues inhibit the growth of efflux-multidrug resistant bacteria and R-plasmids conjugal transfer. *J. Ethnopharmacol.* 245:111871. 10.1016/j.jep.2019.111871 31022566

[B103] Oyedemi B. O. M. Shinde V. Shinde K. Kakalou D. Stapleton P. D. Gibbons S. (2016). Novel R-plasmid conjugal transfer inhibitory and antibacterial activities of phenolic compounds from Mallotus philippensis (Lam.) Mull. *Arg. J. Glob. Antimicrob. Resist* 5 15–21. 10.1016/j.jgar.2016.01.011 27436460

[B104] Ozcan M. (2003). Antioxidant activities of rosemary, sage, and sumac extracts and their combinations on stability of natural peanut oil’. *J. Med. food* 6 267–270. 10.1089/10966200360716698 14585194

[B105] Pagès J.-M. James C. E. Winterhalter M. (2008). The porin and the permeating antibiotic: a selective diffusion barrier in Gram-negative bacteria’. *Nat. Rev. Microbiol.* 6 893–903. 10.1038/nrmicro1994 18997824

[B106] Partridge S. R. Kwong S. M. Firth N. Jensen S. O. (2018). Mobile Genetic Elements Associated with Antimicrobial Resistance. *Clin. Microbiol. Rev.* 31 e00088–17. 10.1128/CMR.00088-17 30068738 PMC6148190

[B107] Patwardhan R. B. Dhakephalkar P. K. Chopade B. A. Dhavale D. D. Bhonde R. R. (2018). Purification and Characterization of an Active Principle, Lawsone, Responsible for the Plasmid Curing Activity of Plumbago zeylanica Root Extracts. *Front. Microbiol.* 9:2618. 10.3389/fmicb.2018.02618 30467495 PMC6236066

[B108] Paul D. Verma J. Banerjee A. Konar D. Das B. (2022). “Antimicrobial Resistance Traits and Resistance Mechanisms in Bacterial Pathogens.” *Antimicrobial Resistance* Kumar V. Shriram V. Paul A. Thakur M. (eds) (Singapore: Springer) 1–27. 10.1007/978-981-16-3120-7_1

[B109] Perlin D. S. Latchney L. R. Senior A. E. (1985). Inhibition of *Escherichia coli* H -ATPase by venturicidin, oligomycin and ossamycin. *Biochim. Biophys. Acta* 807 238–244. 10.1016/0005-2728(85)90254-3 2859888

[B110] Piddock L. (2011). Faculty Opinions recommendation of Combinations of antibiotics and nonantibiotic drugs enhance antimicrobial efficacy. *Faculty Opin.Post-Publicat. Peer Rev. Biomed. Literat.* [Preprint]. 10.3410/f.13348956.14719054

[B111] Ponnusamy K. Ramasamy M. Savarimuthu I. Paulraj M. G. (2010). Indirubin potentiates ciprofloxacin activity in the NorA efflux pump of Staphylococcus aureus. *Scand. J. Infect. Dis.* 42 500–505. 10.3109/00365541003713630 20380543

[B112] Recchia G. D. Hall R. M. (1995). Gene cassettes: a new class of mobile element. *Microbiology* 3015–3027. 10.1099/13500872-141-12-3015 8574395

[B113] Reygaert W. C. Department of Biomedical Sciences, Oakland University William Beaumont School of Medicine, Rochester M. I. Usa (2018). An overview of the antimicrobial resistance mechanisms of bacteria. *AIMS Microbiol.* 4 482–501. 10.3934/microbiol.2018.3.482 31294229 PMC6604941

[B114] Reznikoff W. S. (1993). The TN5 Transposon. *Ann. Rev. Microbiol.* 945–964. 10.1146/annurev.mi.47.100193.004501 7504907

[B115] Rhodes A. Fantes K. H. Boothroyd B. McGONAGLE M. P. Crosse R. (1961). Venturicidin: A New Antifungal Antibiotic of Potential Use in Agriculture. *Nature* 192 952–954. 10.1038/192952a0 14491780

[B116] Santiago C. Pang E. L. Lim K.-H. Loh H.-S. Ting K. N. (2014). Reversal of ampicillin resistance in MRSA via inhibition of penicillin-binding protein 2a by Acalypha wilkesiana. *Biomed Res. Int.* 2014:965348. 10.1155/2014/965348 25101303 PMC4101222

[B117] Schaenzer A. J. Wright G. D. (2020). Antibiotic Resistance by Enzymatic Modification of Antibiotic Targets. *Trends Mol. Med.* 26 768–782. 10.1016/j.molmed.2020.05.001 32493628

[B118] Schindler B. D. Kaatz G. W. (2016). Multidrug efflux pumps of Gram-positive bacteria. *Drug Resist. Updat.* 27 1–13. 10.1016/j.drup.2016.04.003 27449594

[B119] Sengupta M. Austin S. (2011). Prevalence and significance of plasmid maintenance functions in the virulence plasmids of pathogenic bacteria. *Infect. Immunity* 79 2502–2509. 10.1128/IAI.00127-11 21555398 PMC3191983

[B120] Shiota S. Shimizu M. Sugiyama J. ’I. Morita Y. Mizushima T. Tsuchiya T. (2004). Mechanisms of action of corilagin and tellimagrandin I that remarkably potentiate the activity of beta-lactams against methicillin-resistant Staphylococcus aureus. *Microbiol. Immunol.* 48 67–73. 10.1111/j.1348-0421.2004.tb03489.x 14734860

[B121] Shiu W. K. P. Malkinson J. P. Rahman M. M. Curry J. Stapleton P. Gunaratnam M. (2013). A new plant-derived antibacterial is an inhibitor of efflux pumps in Staphylococcus aureus. *Int. J. Antimicrob. Agents* 42 513–518. 10.1016/j.ijantimicag.2013.08.007 24119569

[B122] Shriram V. Jahagirdar S. Latha C. Kumar V. Puranik V. Rojatkar S. (2008). A potential plasmid-curing agent, 8-epidiosbulbin E acetate, from *Dioscorea bulbifera* L. against multidrug-resistant bacteria. *Int. J. Antimicrob. Agents* 32 405–410. 10.1016/j.ijantimicag.2008.05.013 18718743

[B123] Singh K. Naidoo Y. Baijnath H. (2018). A COMPREHENSIVE REVIEW ON THE GENUS PLUMBAGO WITH FOCUS ON *PLUMBAGO AURICULATA* (PLUMBAGINACEAE). *Afr. J. Tradition. Compl. Alternat. Med.* 15 199–215. 10.21010/ajtcam.v15i1.21

[B124] Smith K. P. Kumar S. Varela M. F. (2009). Identification, cloning, and functional characterization of EmrD-3, a putative multidrug efflux pump of the major facilitator superfamily from *Vibrio cholerae* O395. *Arch. Microbiol.* 191 903–911. 10.1007/s00203-009-0521-8 19876617 PMC2809785

[B125] Sousa Silveira Z. de, Macêdo N. S. Sampaio Dos, Santos J. F. Sampaio (2020). Evaluation of the Antibacterial Activity and Efflux Pump Reversal of Thymol and Carvacrol against Staphylococcus aureus and Their Toxicity in Drosophila melanogaster. *Molecules* 25:2103. 10.3390/molecules25092103 PMC7249103 32365898

[B126] Stapleton P. Wu P. J. King A. Shannon K. French G. Phillips I. (1995). Incidence and mechanisms of resistance to the combination of amoxicillin and clavulanic acid in *Escherichia coli*. *Antimicrobial Agents Chemother.* 39 2478–2483. 10.1128/aac.39.11.2478 PMC162968 8585729

[B127] Stermitz F. R. Lorenz P. Tawara J. N. Zenewicz L. A. Lewis K. (2000). Synergy in a medicinal plant: antimicrobial action of berberine potentiated by 5’-methoxyhydnocarpin, a multidrug pump inhibitor. *Proc. Natl. Acad. Sci. U.S.A.* 97 1433–1437. 10.1073/pnas.030540597 10677479 PMC26451

[B128] Stokes H. W. Hall R. M. (1989). A novel family of potentially mobile DNA elements encoding site-specific gene-integration functions: integrons. *Mol. Microbiol.* 3 1669–1683. 10.1111/j.1365-2958.1989.tb00153.x 2560119

[B129] Sudano Roccaro A. Blanco A. R. Giuliano F. Rusciano D. Enea V. (2004). Epigallocatechin-gallate enhances the activity of tetracycline in staphylococci by inhibiting its efflux from bacterial cells. *Antimicrob. Agents Chemother.* 48 1968–1973. 10.1128/AAC.48.6.1968-1973.2004 15155186 PMC415601

[B130] Sun J. Deng Z. Yan A. (2014). Bacterial multidrug efflux pumps: mechanisms, physiology and pharmacological exploitations. *Biochem. Biophys. Res. Commun.* 453 254–267. 10.1016/j.bbrc.2014.05.090 24878531

[B131] Thiara A. S. Cundliffe E. (1988). Cloning and characterization of a DNA gyrase B gene from Streptomyces sphaeroides that confers resistance to novobiocin. *EMBO J.* 7 2255–2259. 10.1002/j.1460-2075.1988.tb03065.x 2843361 PMC454581

[B132] Toda M. Okubo S. Hara Y. Shimamura T. (1991). Antibacterial and bactericidal activities of tea extracts and catechins against methicillin resistant Staphylococcus aureus. *Nippon Saikingaku Zasshi* 46 839–845. 10.3412/jsb.46.839 1762174

[B133] Upadhyay H. C. Dwivedi G. R. Darokar M. P. Chaturvedi V. Srivastava S. K. (2012). Bioenhancing and antimycobacterial agents from Ammannia multiflora. *Planta Med.* 78 79–81. 10.1055/s-0031-1280256 21969115

[B134] Upadhyay H. C. Thakur J. P. Saikia D. Srivastava S. K. (2013). Anti-tubercular agents from Ammannia baccifera (Linn.). *Med. Chem. Res.* 22 16–21. 10.1007/s00044-012-9998-9

[B135] Vaara M. (2019). Polymyxin Derivatives that Sensitize Gram-Negative Bacteria to Other Antibiotics. *Molecules* 24:249. 10.3390/molecules24020249 PMC6359160 30641878

[B136] Vemuri P. K. Dronavalli L. Nayakudugari P. Kunta A. Challagulla R. (2019). Phytochemical Analysis and Biochemical Characterization of Terminalia Chebula Extracts For its Medicinal use. *Biomed. Pharmacol. J.* 12 1525–1529. 10.13005/bpj/1783

[B137] Ventola C. L. (2015). The antibiotic resistance crisis: part 1: causes and threats. *P T* 40 277–283. 25859123 PMC4378521

[B138] Verma P. Tiwari M. Tiwari V. (2021). Efflux pumps in multidrug-resistant *Acinetobacter baumannii*: Current status and challenges in the discovery of efflux pumps inhibitors. *Microbial Pathogenesis* 152:104766. 10.1016/j.micpath.2021.104766 33545327

[B139] Wang D. Xie K. Zou D. Meng M. Xie M. (2018). Inhibitory effects of silybin on the efflux pump of methicillin-resistant Staphylococcus aureus. *Mol. Med. Rep.* 18 827–833. 10.3892/mmr.2018.9021 29845191 PMC6059712

[B140] Wang Y. Alenzy R. Song D. Liu X. Teng Y. Mowla R. (2020). Structural optimization of natural product nordihydroguaretic acid to discover novel analogues as AcrB inhibitors. *Eur. J. Med. Chem.* 186:111910. 10.1016/j.ejmech.2019.111910 31801655

[B141] Webber M. A. (2003). The importance of efflux pumps in bacterial antibiotic resistance. *Journal of Antimicrobial Chemotherapy* 51 9–11. 10.1093/jac/dkg050 12493781

[B142] Willyard C. (2017). The drug-resistant bacteria that pose the greatest health threats. *Nature* 543:15. 10.1038/nature.2017.21550 28252092

[B143] Wozniak R. A. F. Waldor M. K. (2010). Integrative and conjugative elements: mosaic mobile genetic elements enabling dynamic lateral gene flow. *Nat. Rev. Microbiol.* 8 552–563. 10.1038/nrmicro2382 20601965

[B144] Wright G. D. (2016). Antibiotic Adjuvants: Rescuing Antibiotics from Resistance: (Trends in Microbiology 24, 862-871; October 17, 2016). *Trends Microbiol.* 24 928. 10.1016/j.tim.2016.07.008 27430191

[B145] Xu J. Jia H. Cui G. Tong H. Wei J. Shao D. (2018). ICEAplChn1, a novel SXT/R391 integrative conjugative element (ICE), carrying multiple antibiotic resistance genes in Actinobacillus pleuropneumoniae. *Vet. Microbiol.* 220 18–23. 10.1016/j.vetmic.2018.05.002 29885796

[B146] Yarlagadda V. Medina R. Wright G. D. (2020). Venturicidin A, A Membrane-active Natural Product Inhibitor of ATP synthase Potentiates Aminoglycoside Antibiotics. *Sci. Rep.* 10:8134. 10.1038/s41598-020-64756-0 32424122 PMC7235042

[B147] Zhao W.-H. Hu Z.-Q. Okubo S. Hara Y. Shimamura T. (2001). Mechanism of Synergy between Epigallocatechin Gallate and β-Lactams against Methicillin-Resistant Staphylococcus aureus. *Antimicrobial Agents Chemother.* 45 1737–1742. 10.1128/aac.45.6.1737-1742.2001 PMC90539 11353619

[B148] Zhou X.-Z. Jia F. Liu X.-M. Yang C. Zhao L. Wang Y.-J. (2013). Total alkaloids from Sophora alopecuroides L. increase susceptibility of extended-spectrum β-lactamases producing *Escherichia coli* isolates to cefotaxime and ceftazidime. *Chin. J. Integr. Med.* 19 945–952. 10.1007/s11655-011-0899-4 22528755

