# Supplementary material for: Antibiotic Potentiators Against Multidrug-Resistant Bacteria: Discovery, Development, and Clinical Relevance
Source: Front Microbiol. 2022 Jul 1;13:887251. doi: 10.3389/fmicb.2022.887251 (PMC9284026; doi:10.3389/fmicb.2022.887251)
Supplement: Supplementary file 1 [file Data_Sheet_1.PDF]

Supplementary Table 1: Antibiotic resistance breakers targeting different modes of AMR

Synthetic and natural compounds augmenting the activity of the antibiotics and their current clinical status.

S.No	Inhibitor type	Compound name	Augmented antibiotics	Targets	Current status	Isolation source	References
	**β -lactamase inhibitors (BLIs)**						
**1.**	Fungal-derived	Clavulanic acid	Amoxicillin, Ticarcillin	Class A narrow spectrum, Class A ESBLs	Trials completed, marketed as Augmentin®, Timentin®	*Streptomyces clavuligerus*	Bush, 1988; Laws, Shaaban and Rahman, 2019
**2.**	Penicillin-based sulfones	Sulbactam	Amoxicillin, Cefoperazone, Ampicillin, Piperacillin	Class A narrow spectrum, Class A ESBLs, TEM-type β-lactamases, SHV- and OXA-type β-lactamases	Trials completed, marketed as Unasyn®	Synthetic	Adnan *et al.*, 2013; Penwell *et al.*, 2015
**3.**		Tazobactam	Piperacillin, Ceftolozane	Class A narrow spectrum, Class A ESBLs Some Class C enzymes TEM-type β-lactamases, SHV- and OXA-type β-lactamases	Trials completed, marketed as Zosyn®	Synthetic	Bush, 2015
**4.**	Diazabicyclooctane (DABCO)	Avibactam	Ceftaroline, Ceftazidime, Aztreonam	Class A narrow spectrum, Class A ESBLs, Class A carbapenemases, Class C and D enzymes, TEM-1, KPC-2 and SHV-4	Trials completed, marketed as Avycaz®, Zavicefta®	Synthetic	Bonnefoy *et al.*, 2004; Ehmann *et al.*, 2012
**5.**		Relebactam (MK-7655)	Imipenem	Class A narrow spectrum, Class A ESBLs, Class A carbapenemases, Some Class C enzymes	Trials completed, marketed as Recarbrio®	Synthetic	Blizzard *et al.*, 2014
**6.**		Zidebactam (WCK 5222)	Cefepime	Inhibits class A and C β-lactamases and PBP-2 of *K. pneumoniae*, *P. aeruginosa*, and *A. baumannii* and also	Undergoing a phase III clinical trial	Synthetic	Livermore *et al.*, 2017

				inhibits Enterobacteriaceae producing metallo-β-lactamases and class D oxacillinases and *P. aeruginosa* with metallo-β-lactamases			
**7.**	Boronic acid inhibitor	Vaborbactam	Biapenem, Meropenem, Doripenem, Ertapenem	Broad spectrum of class A, C and D enzymes, including KPC, CTX-M, SHV, and CMY	Completed phase III trials, marketed as Vabomere™, Carbavance®	Synthetic	Hecker *et al.*, 2015
**8.**		Taniborbactam (VNRX-5133)	Cefepime	Class A, C and D serine β lactamases and VIM/NDM, Class B MBLs in both carbapenem-resistant Enterobacteriaceae and *P. aeruginosa*	Completed phase III clinical trials.	Synthetic	Hackel and Sahm, 2018; Daigle *et al.*, 2018
**9.**		QPX7728	Multiple β -lactam antibiotics such as Meropenem cefepime and aztreonam, ceftazidime	Inhibits serine- and metallo-β - lactamases of Classes A, B, C, and D in *Acinetobacter* spp., *Pseudomonas aeruginosa*, and Enterobacteriaceae	Clinical development initiated	Synthetic	Lomovskaya *et al.* 202[illegible]
**10.**	Metallo-beta-lactamases (MBL) inhibitors	ANT 431	Meropenem	MBLs and MBL-producing carbapenem resistant *Enterobacteriaceae* (CRE)	Undergoing clinical trials	Synthetic	Everett *et al.*, 2018; Leiris *et al.*, 2019
**11.**		Magnolol	Meropenem	New Delhi metallo-β-lactamase (NDM)	Undergoing clinical trials	*Magnolia officinalis*	Liu *et al.*, 2018; Zhang *et al.*, 2019
**12.**		Aspergillomarasmine A	Meropenem	NDM-1 enzyme and other MBLs, VIM-2	Undergoing clinical trials	*Aspergillus versicolor*	Gupta and Datta, 2019; King *et al.*, 2014
**Inhibitors of antibiotic resistance enzymes**							
**13.**	Protein kinase inhibitors	Pyrazolopyrimidine 2a, CKI-7, quercetin, damnacanthal, wortmannin	Various aminoglycosides in Gram negative bacteria	aminoglycoside O-phosphotransferases (APHs) including, APH (3') -Ia, APH (2′′) -Ia	Lab study	Synthetic	Stogios *et al.*, 2013
**14.**	Bisubstrate analogues	Kanamycin B-CoA	Kanamycin	AAC(6′)-Ii	No clinical trials yet	Synthetic	Gao *et al.*, 2005
**15.**		Gentamicin-CoA	Gentamycin	AAC(3)-I	No clinical trials yet	Synthetic	Gao *et al.*, 2005

**16.**	Oxidopyrylium cyclo addition/ring opening strategy inhibitors	α-hydroxytropolones 3e and 3o	Gentamicin	ANT(2″)-Ia	No clinical trials yet	Synthetic	Hirsch *et al.*, 2014
**17.**	Bovine antimicrobial peptide	Indolicidin	Various aminoglycosides	Members of AACs and APHs families	No clinical trials yet	Synthetic	Boehr *et al.*, 2003
**18.**	Fungal-derived	Aranorosin	Various aminoglycosides	AAC(6′)Ie and APH(2″)-Ia	No clinical trials yet	*Gymnascella aurantiaca*	Suga *et al.*, 2012
**19.**	Nucleotide-neamine complex	Adenosine-neamine complex	Various aminoglycosides	APHs and ANTs enzyme classes	No clinical trials yet	Synthetic	Liu, Haddad, *et al.*, 2000
**20.**	As decoy acceptor	Streptidine	Streptomycin	AME 6-O-adenyl transferase	Lab study	Synthetic	Latorre *et al.*, 2007
**Efflux pump inhibitors (EPIs)**							
**21.**	Inhibition by competitive or non- competitive direct binding to functional efflux pumps	phenylalanine-arginine β-naphthylamide (PaβN) or MC-207,110	Levofloxacin, erythromycin, chloramphenicol, clindamycin and trimethoprim in gram negative bacteria	RND, AdeFGH pumps,	Toxic in nature, confined to labs	Synthetic	Vargiu and Nikaido, 2012
**22.**		Verapamil	Bedaquiline and ofloxacin in *M. tuberculosis*.	MATE pumps DinF and NorM,	Toxic in nature, confined to labs	Synthetic	Gupta *et al.*, 2014
**23.**		1-(1-naphthylmethyl)-piperazine (NMP)	Levofloxacin, oxacillin, rifampin, chloramphenicol, clarithromycin, fluoroquinolones, azithromycin, clindamycin, nitrofurantoin and doxycycline	AdeABC, AcrAB, AcrEF pumps in *A. baumannii, E. coli, Enterobacter aerogenes, K. pneumonia*	Lab study	Synthetic	Vargiu *et al.*, 2014
**24.**	Based on energy dissipation: Efflux inhibition carried out by the decoupling of energy and efflux	Carbonyl cyanide-m-chlorophenylhydrazone (CCCP)	Tetracycline in *Helicobacter pylori* and *Klebsiella* spp. and carbapenems	Disrupts the proton motive force (PMF) by affecting Δψ and ΔpH	Toxic, confined to labs	Synthetic	Anoushiravani, Falsafi and Niknam, 2009; Fenosa *et al.*, 2009

**25.**	activity	IITR08027	Ciprofloxacin, fluoroquinolones in *E. coli* and *A. baumannii*	MATE efflux pump AbeM by disturbing its proton gradient	No clinical trials yet	Synthetic	Bhattacharyya *et al.*, 2017
**26.**	Quinoline derivatives	Pyridoquinolones, 2-phenyl-4(1H)-quinolone and 2-phenyl-4-hydroxyquinoline	Norfloxacin in *E. aerogenes*	RND AcrAB-TolC efflux pump, NorA efflux pump in *S. aureus*	Lab study	Synthetic	Chevalier *et al.*, 2001
**27.**	Chalcones derivatives		Norfloxacin in *S. aureus* SA1199B	NorA inhibitor	Lab study	Synthetic	Alves Borges Leal *et al.*, 2021
**28.**	Arylpiperidines and aryl piperazine derivatives	Arylpiperidine and phenylpiperidines	Linezolid	MDR efflux pumps in *S. aureus*, AcrAB-TolC pump in *E. coli*	Lab study	Synthetic	Thorarensen *et al.*, 2001; Kaatz *et al.*, 200[illegible]
**29.**	Pyridopyrimidine and pyranopyridine derivatives	Pyridopyrimidines : D2 and D13-9001	Fluoroquinolones	MexAB-OprM-specific pump in *P. aeruginosa* and AcrB in *E. coli*	Lab study	Synthetic	Mahmood *et al.*, 2016
**30.**		MBX2319	Ciprofloxacin, levofloxacin and piperacillin	AcrAB pump in *E. coli*	Lab study	Synthetic	Vargiu *et al.*, 2014
**31.**	Thieno[3,2-*c*]pyran-2-ones	3-amino-2-carbomethoxy-6-aryl and 5,6-diaryl-4*H*-thieno[3,2-*c*]pyran-2-ones	Norfloxacin, ofloxacin, tobramycin, Amikacin, ampicillin, cefoxitin, cefotaxime, oxy-tetracycline & tetracycline	MexAB-OprM pump in *P. Aeruginosa*	Lab study	Synthetic	Dwivedi *et al.*, 2021
**32.**	Heterocyclic Carboxamides	TXA01182	Monobactam, fluoroquinolones, sulfonamide and tetracyclines	Efflux pump inhibitor	Lab study	Synthetic	Yuan *et al.*, 2021
**33.**	Plant alkaloids	Reserpine	Tetracycline in *B. subtilis*, norfloxacin in *S. aureus*	MFS and RND pumps superfamily	Lab study	*Rauwolfia serpentina*	Stavri, Piddock and Gibbons, 2007
**34.**		Piperine	Ciprofloxacin in *S. aureus* rifampicin in *M.*	Human P-glycoprotein of ABC transporters, NorA efflux pump in *S. aureus*	Lab study	*Piper nigram*	Sharma *et al.*, 2010

			tuberculosis H37Rv and ethidium bromide in *M. smegmatis*				
**35.**	Plant-derived flavonoids	Baicalein	Ciprofloxacin and β-lactam antibiotics including oxacillin, cefmetazole and ampicillin and tetracycline	NorA, TetK efflux pumps	Phase II clinical trial	*Thymus vulgaris*	Chan *et al.*, 2011 ;Fujita *et al.*, 200[illegible]
**36.**		5'-methoxy-hydnocarpin	Norfloxacin and berberine	NorA pump	Lab study	*Berberis fremontii*	Stermitz *et al.*, 2000
**37.**		Genistein, orobol and biochanin A	Norfloxacin and berberine in *M. smegmatis* and *S. aureus*	NorA, MDR efflux pumps	Lab study	*Lupinus argenteus*	Morel *et al.,* 2003
**38.**	Polyphenols	Catechin gallates including epicatechin gallate and Epigallocatechin gallate	Tetracycline, erythromycin and ciprofloxacin in *Staphylococci* and *Campylobacter* spp, reverse MRSA resistance	NorA efflux pump<br><br>Tet(K)efflux pump	Lab study	*Camellia sinensis*	Gibbons, 2004
**39.**	Phenolic diterpenes	Carnosic acid and carnosol	Tetracycline and erythromycin against macrolide-resistant *S. aureus*	ABC transporters, MsrA and TetK efflux pumps	Lab study	*Rosmarinus officinalis*	Oluwatuyi, Kaatz and Gibbons, 2004
**40.**		Geraniol	Chloramphenicol	MDR efflux, tripartite AcrAB-TolC pump in *Enterobacter aerogenes* CM-64,	Lab study	*Helichrysum italicum*	Lorenzi *et al.*, 2009
**41.**	Derived from microbes	EA-371α and EA-371δ	Levofloxacin	MexAB-OprM pump in *P. Aeruginosa*	Lab study	Fermentation extract of *Streptomyces* sp p	Lee *et al.*, 2001
**42.**		Isovaleryl shikonin (IVS)	Streptomycin	MsrA efflux pump	Lab study	*Arnebia euchroma*	He *et al.*, 2019
**43.**		methyl-1α-acetoxy-7α 14α-	Erythromycin, tetracycline	Tet(K) Msr(A) efflux pump	Lab study	*Lycopus europaeus*	Gibbons *et al.*, 2003

		dihydroxy-8,15-isopimaradien-18-oate<br><br>methyl-1α,14α-diacetoxy-7α-hydroxy- 8,15-isopimaradien-18-oate					
**44.**		2,6-dimethyl-4-phenyl-pyridine-3,5-dicarboxylic acid diethyl ester	Ciprofloxacin, norfloxacin	NorA efflux pump	Lab study	*J. elliptica*	Marquez *et al.*, 2005
**45.**	Derived from plants	kaempferol-3-*O*-α-L-(2,4-bis-*E*-*p*-coumaroyl)rhamnoside	Ciprofloxacin	NorA efflux pump	Lab study	*Persea lingue*	Holler *et al.*, 2012
**46.**		Indirubin	Ciprofloxacin	NorA efflux pump	Lab study	*Wrightia tinctoria*	Ponnusamy *et al.*, 201[illegible]
**47.**		Capsaicin	Ciprofloxacin	NorA efflux pump	Lab study	*Capsicum annum*	Kalia *et al.*, 2012
**48.**		Olympicin A	Enoxacin	NorA efflux pump	Lab study	*Hypericum olympicum*	Shiu *et al.*, 2013
**49.**		4□hydroxy□*α*□tetralone	Tetracycline	ATP dependent efflux pumps	Lab study	*Ammannia multiflora*	Upadhyay *et al., 2012*
**50.**		Silybin	Ciprofloxacin	Quinolone resistance protein NorA (*norA*) and proteins A/B (*qacA/B*) efflux genes	Lab study	Milk thistle seed	Wang *et al.*, 2018
**51.**		*Punica granatum* methanolic extract (PGME)	Chlorampheniol, ampicillin, gentamicin, oxacillin, and tetracycline	NorA efflux pump	Lab study	*Punica granatum*	Braga *et al.,* 2005
**52.**		Extract and component allyl sulphide	Chloramphenicol, linezolid, erythromycin, minocycline, rifampin, rhodamine 6G, trimethoprim and	EmrD-3 efflux	Lab study	*Allium sativum*	Bruns *et al.*, 2017

			tetraphenylphosphonium chloride				
**Membrane permeabilizers**							
**53.**	The polymyxins and its derivatives	Polymyxin B, Polymyxin E (colistin)	Azithromycin, rifampicin	Mg^{2+} and Ca^{2+} ions displacement from bacterial outer membrane	Used in clinical settings	Synthetic	Lee *et al.*, 2013; Lin *e[illegible]* *al.*, 2015
**54.**		Polymyxin B nonapeptide (PMBN)	Erythromycin, clindamycin, rifampicin, fusidic acid, novobiocin, cloxacillin, ampicillin, lincomycin, nafcillin and vancomycin	Mg^{2+} and Ca^{2+} ions displacement from bacterial outer membrane	Used in clinical settings	Synthetic	Vaara and Vaara, 198[illegible]
**55.**		NAB739 and NAB741	Rifampicin, vancomycin and clarithromycin, azithromycin, dalfopristin, erythromycin, fidaxomicin, fosfomycin, fusidic acid, mupirocin, novobiocin, ramoplanin, retapamulin, telithromycin	Cationic destabilization of bacterial OM	Phase I clinical trials (as SPR741)	Synthetic	Vaara, 2009; Vaara *et al.*, 2010; Eckburg *et al.*, 2019
**56.**	Lysine-based peptidomimetics	H-[NLys-tBuAla]6-NH2 (CEP-136)	Rifampicin, clarithromycin, and azithromycin	Permeabilization effect on OM	Lab study	Synthetic	Mood *et al.*, 2021
**57.**	Macromolecular potentiator	WD40	Rifampin	Permeabilization of membrane	Lab study	Synthetic	Chan *et al.*, 2021
**58.**	~140 000 diverse synthetic compounds	Liproxstatin-1 and MAC-0568743	Rifampicin, novobiocin and erythromycin,	Disrupt the integrity of the OM	Lab study	Synthetic	Klobucar *et al.*, 2021

			linezolid.				
**59.**	Meldrum's acid derivative	N-{6-[(2,2-Dimethyl-4,6-dioxo-[1,3]dioxane-5-ylidenomethyl)-amino]-pyridin-2-yl}-acetamide (MAD)	Norfloxacin, ofloxacin and lomefloxacin	Not sudied	Lab study	Synthetic	da Silva *et al.*, 2021
**60.**	Chalcone and derivates	chalcone (E)-1-(4-aminophenyl)-3-(furan-2-yl)-prop-2-en-1-one (C13H11NO2) (AFPO)	Norfloxacin, penicillin, ampicillin/sulbactam and gentamicin	Not studied	Lab study	Synthetic	Ferraz *et. al*, 2020
**61.**	Phytochemicals	Gallic acid and thymol	23 antibiotics including azithromycin, erythromycin, nitrofurantoin, novobiocin, sulfamethoxazole, trimethoprim etc.	Membrane destabilisation	Lab study	Natural	Farrag *et al.*, 2019
**62.**	Citrus limon (EOCL) and Cinnamomum zeylanicum (EOCZ)	Essential oil	Amikacin, imipenem and meropenem.	Not studied	Lab study	Natural	Guerra *et al.*, 2012
**63.**	*Albizia lebbeck*, *Baillonella toxisperma, Nauclea pobeguinii, Aframomum sulcatum*	Methanol extract and seed hydrolate	Amoxicillin, ampicillin, ceftriaxone, and norfloxacin	Not studied	Lab study	Natural	Njimoh *et al.*, 2015
**64.**	NCI Natural Products Set IV library	Clorobiocin, prodigiosin, and novobiocin	Aminoglycosides, β-lactams, polymyxins, and macrolides.	Not studied	Lab study	Natural	Mattingly *et al.*, 2020

References

1. Adnan, S. *et al.* (2013) 'Ampicillin/sulbactam: its potential use in treating infections in critically ill patients', *International journal of antimicrobial agents*, 42(5), pp. 384–389.
2. Alves Borges Leal, A.L. *et al.* (2021) 'Potentiating activity of Norfloxacin by synthetic chalcones against NorA overproducing Staphylococcus aureus', *Microbial pathogenesis*, 155, p. 104894.
3. Anoushiravani, M., Falsafi, T. and Niknam, V. (2009) 'Proton motive force-dependent efflux of tetracycline in clinical isolates of Helicobacter pylori', *Journal of medical microbiology*, 58(Pt 10), pp. 1309–1313.
4. Bhattacharyya, T. *et al.* (2017) 'The small molecule IITR08027 restores the antibacterial activity of fluoroquinolones against multidrug-resistant Acinetobacter baumannii by efflux inhibition', *International journal of antimicrobial agents*, 50(2), pp. 219–226.
5. Blizzard, T.A. *et al.* (2014) 'Discovery of MK-7655, a β-lactamase inhibitor for combination with Primaxin®', *Bioorganic & medicinal chemistry letters*, 24(3), pp. 780–785.
6. Boehr, D.D. *et al.* (2003) 'Broad-spectrum peptide inhibitors of aminoglycoside antibiotic resistance enzymes', *Chemistry & biology*, pp. 189–196.
7. Bonnefoy, A. *et al.* (2004) 'In vitro activity of AVE1330A, an innovative broad-spectrum non-beta-lactam beta-lactamase inhibitor', *The Journal of antimicrobial chemotherapy*, 54(2), pp. 410–417.
8. Braga, L.C. *et al.* (2005) 'Synergic interaction between pomegranate extract and antibiotics against Staphylococcus aureus', *Canadian journal of microbiology*, 51(7), pp. 541–547.
9. Bruns, M.M. *et al.* (2017) 'Modulation of the multidrug efflux pump EmrD-3 from Vibrio cholerae by Allium sativum extract and the bioactive agent allyl sulfide plus synergistic enhancement of antimicrobial susceptibility by A. sativum extract', *Archives of microbiology*, 199(8), pp. 1103–1112.

10. Bush, K. (1988) ‘Beta-lactamase inhibitors from laboratory to clinic’, *Clinical Microbiology Reviews*, pp. 109–123. doi:10.1128/cmr.1.1.109-123.1988.

11. Bush, K. (2015) ‘A resurgence of β-lactamase inhibitor combinations effective against multidrug-resistant Gram-negative pathogens’, *International journal of antimicrobial agents*, 46(5), pp. 483–493.

12. Chan, B.C.L. *et al.* (2011) ‘Synergistic effects of baicalein with ciprofloxacin against NorA over-expressed methicillin-resistant Staphylococcus aureus (MRSA) and inhibition of MRSA pyruvate kinase’, *Journal of ethnopharmacology*, 137(1), pp. 767–773.

13. Chan, L.W. *et al.* (2021) ‘Selective Permeabilization of Gram-Negative Bacterial Membranes Using Multivalent Peptide Constructs for Antibiotic Sensitization’, *ACS infectious diseases*, 7(4), pp. 721–732.

14. Chevalier, J. *et al.* (2001) ‘New Pyridoquinoline Derivatives as Potential Inhibitors of the Fluoroquinolone Efflux Pump in Resistant Enterobacter aerogenes Strains’, *Journal of Medicinal Chemistry*, pp. 4023–4026. doi:10.1021/jm010911z.

15. Daigle, D. *et al.* (2018) ‘1370. Cefepime/VNRX-5133 Broad-Spectrum Activity Is Maintained Against Emerging KPC- and PDC-Variants in Multidrug-Resistant K. pneumoniae and P. aeruginosa’, *Open Forum Infectious Diseases*, pp. S419–S420. doi:10.1093/ofid/ofy210.1201.

16. Dwivedi, G.R. *et al.* (2021) ‘Drug resistance reversal potential of multifunctional thieno[3,2-c]pyran via potentiation of antibiotics in MDR P. aeruginosa’, *Biomedicine & pharmacotherapy = Biomedecine & pharmacotherapie*, 142, p. 112084.

17. Eckburg, P.B. *et al.* (2019) ‘Safety, Tolerability, Pharmacokinetics, and Drug Interaction Potential of SPR741, an Intravenous Potentiator, after Single and Multiple Ascending Doses and When Combined with β-Lactam Antibiotics in Healthy Subjects’, *Antimicrobial agents and chemotherapy*, 63(9). doi:10.1128/AAC.00892-19.

18. Ehmann, D.E. *et al.* (2012) ‘Avibactam is a covalent, reversible, non-β-lactam β-lactamase inhibitor’, *Proceedings of the National Academy of Sciences of the United States of America*, 109(29), pp. 11663–11668.

19. Everett, M. *et al.* (2018) ‘Discovery of a Novel Metallo-β-Lactamase Inhibitor That Potentiates Meropenem Activity against Carbapenem-Resistant Enterobacteriaceae’, *Antimicrobial agents and chemotherapy*, 62(5). doi:10.1128/AAC.00074-18.
20. Farrag, H.A. *et al.* (2019) ‘Natural outer membrane permeabilizers boost antibiotic action against irradiated resistant bacteria’, *Journal of biomedical science*, 26(1), p. 69.
21. Fenosa, A. *et al.* (2009) ‘Role of TolC in Klebsiella oxytoca resistance to antibiotics’, *The Journal of antimicrobial chemotherapy*, 63(4), pp. 668–674.
22. Fujita, M. *et al.* (2005) ‘Remarkable synergies between baicalein and tetracycline, and baicalein and beta-lactams against methicillin-resistant Staphylococcus aureus’, *Microbiology and immunology*, 49(4), pp. 391–396.
23. Gao, F. *et al.* (2005) ‘Regio- and chemoselective 6’-N-derivatization of aminoglycosides: bisubstrate inhibitors as probes to study aminoglycoside 6'-N-acetyltransferases’, *Angewandte Chemie* , 44(42), pp. 6859–6862.
24. Gibbons, S. *et al.* (2003) ‘Bacterial resistance modifying agents from Lycopus europaeus’, *Phytochemistry*, 62(1), pp. 83–87.
25. Gibbons, S., Moser, E. and Kaatz, G.W. (2004) ‘Catechin gallates inhibit multidrug resistance (MDR) in Staphylococcus aureus’, *Planta medica*, 70(12), pp. 1240–1242.
26. Guerra, F.Q.S. *et al.* (2012) ‘Increasing antibiotic activity against a multidrug-resistant Acinetobacter spp by essential oils of Citrus limon and Cinnamomum zeylanicum’, *Natural product research*, 26(23), pp. 2235–2238.
27. Gupta, S. *et al.* (2014) ‘Efflux inhibition with verapamil potentiates bedaquiline in Mycobacterium tuberculosis’, *Antimicrobial agents and chemotherapy*, 58(1), pp. 574–576.
28. Gupta, V. and Datta, P. (2019) ‘Next-generation strategy for treating drug resistant bacteria: Antibiotic hybrids’, *The Indian journal of medical research*, 149(2), pp. 97–106.
29. Hackel, M. and Sahm, D. (2018) ‘1360. Antimicrobial Activity of Cefepime in Combination with VNRX-5133 Against a Global Collection of Enterobacteriaceae Including Resistant Phenotypes’, *Open Forum Infectious Diseases*, pp. S416–S417. doi:10.1093/ofid/ofy210.1191.

30. Hecker, S.J. *et al.* (2015) ‘Discovery of a Cyclic Boronic Acid β-Lactamase Inhibitor (RPX7009) with Utility vs Class A Serine Carbapenemases’, *Journal of Medicinal Chemistry*, pp. 3682–3692. doi:10.1021/acs.jmedchem.5b00127.
31. He, J.-M. *et al.* (2019) ‘Isovalerylshikonin, a new resistance-modifying agent from Arnebia euchroma, supresses antimicrobial resistance of drug-resistant Staphylococcus aureus’, *International journal of antimicrobial agents*, 53(1), pp. 70–73.
32. Hirsch, D.R. *et al.* (2014) ‘Inhibition of the ANT(2")-Ia resistance enzyme and rescue of aminoglycoside antibiotic activity by synthetic α-hydroxytropolones’, *Bioorganic & medicinal chemistry letters*, 24(21), pp. 4943–4947.
33. Holler, J.G. *et al.* (2012) ‘Novel inhibitory activity of the Staphylococcus aureus NorA efflux pump by a kaempferol rhamnoside isolated from Persea lingue Nees’, *The Journal of antimicrobial chemotherapy*, 67(5), pp. 1138–1144.
34. Kaatz, G.W. *et al.* (2003) ‘Phenylpiperidine selective serotonin reuptake inhibitors interfere with multidrug efflux pump activity in Staphylococcus aureus’, *International journal of antimicrobial agents*, 22(3), pp. 254–261.
35. Kalia, N.P. *et al.* (2012) ‘Capsaicin, a novel inhibitor of the NorA efflux pump, reduces the intracellular invasion of Staphylococcus aureus’, *The Journal of antimicrobial chemotherapy*, 67(10), pp. 2401–2408.
36. King, A.M. *et al.* (2014) ‘Aspergillomarasmine A overcomes metallo-β-lactamase antibiotic resistance’, *Nature*, 510(7506), pp. 503–506.
37. Klobucar, K. *et al.* (2021) ‘Chemical Screen for Vancomycin Antagonism Uncovers Probes of the Gram-Negative Outer Membrane’, *ACS chemical biology*, 16(5), pp. 929–942.
38. Latorre, M. *et al.* (2007) ‘Rescue of the streptomycin antibiotic activity by using streptidine as a “decoy acceptor” for the aminoglycoside-inactivating enzyme adenyl transferase’, *Chemical communications* , (27), pp. 2829–2831.
39. Laws, M., Shaaban, A. and Rahman, K.M. (2019) ‘Antibiotic resistance breakers: current approaches and future directions’, *FEMS microbiology reviews*, 43(5), pp. 490–516.

40. Lee, H.J. *et al.* (2013) ‘Synergistic activity of colistin and rifampin combination against multidrug-resistant Acinetobacter baumannii in an in vitro pharmacokinetic/pharmacodynamic model’, *Antimicrobial agents and chemotherapy*, 57(8), pp. 3738–3745.
41. Lee, M.D. *et al.* (2001) ‘Microbial fermentation-derived inhibitors of efflux-pump-mediated drug resistance’, *Farmaco* , 56(1-2), pp. 81–85.
42. Leiris, S. *et al.* (2019) ‘SAR Studies Leading to the Identification of a Novel Series of Metallo-β-lactamase Inhibitors for the Treatment of Carbapenem-Resistant Enterobacteriaceae Infections That Display Efficacy in an Animal Infection Model’, *ACS Infectious Diseases*, pp. 131–140. doi:10.1021/acsinfecdis.8b00246.
43. Lin, L. *et al.* (2015) ‘Azithromycin Synergizes with Cationic Antimicrobial Peptides to Exert Bactericidal and Therapeutic Activity Against Highly Multidrug-Resistant Gram-Negative Bacterial Pathogens’, *EBioMedicine*, 2(7), pp. 690–698.
44. Liu, M., Haddad, J., *et al.* (2000) ‘Tethered bisubstrate derivatives as probes for mechanism and as inhibitors of aminoglycoside 3’-phosphotransferases’, *The Journal of organic chemistry*, 65(22), pp. 7422–7431.
45. Liu, M., Kirpekar, F., *et al.* (2000) ‘The tylosin resistance gene tlrB of Streptomyces fradiae encodes a methyltransferase that targets G748 in 23S rRNA’, *Molecular microbiology*, 37(4), pp. 811–820.
46. Liu, S. *et al.* (2018) ‘Magnolol restores the activity of meropenem against NDM-1-producing Escherichia coli by inhibiting the activity of metallo-beta-lactamase’, *Cell Death Discovery*. doi:10.1038/s41420-018-0029-6.
47. Livermore, D.M. *et al.* (2017) ‘In vitro activity of cefepime/zidebactam (WCK 5222) against Gram-negative bacteria’, *Journal of Antimicrobial Chemotherapy*, pp. 1373–1385. doi:10.1093/jac/dkw593.
48. Lomovskaya, O. *et al.* (2021) ‘QPX7728, An Ultra-Broad-Spectrum B-Lactamase Inhibitor for Intravenous and Oral Therapy: Overview of Biochemical and Microbiological Characteristics’, *Frontiers in microbiology*, 12, p. 697180.
49. Lorenzi, V. *et al.* (2009) ‘Geraniol restores antibiotic activities against multidrug-resistant isolates from gram-negative species’, *Antimicrobial agents and chemotherapy*, 53(5), pp. 2209–2211.

50. Mahmood, H.Y. *et al.* (2016) ‘Current Advances in Developing Inhibitors of Bacterial Multidrug Efflux Pumps’, *Current medicinal chemistry*, 23(10), pp. 1062–1081.
51. Marquez, B. *et al.* (2005) ‘Multidrug resistance reversal agent from Jatropha elliptica’, *Phytochemistry*, 66(15), pp. 1804–1811.
52. Mattingly, A.E. *et al.* (2020) ‘Screening an Established Natural Product Library Identifies Secondary Metabolites That Potentiate Conventional Antibiotics’, *ACS infectious diseases*, 6(10), pp. 2629–2640.
53. Mood, E.H. *et al.* (2021) ‘Antibiotic Potentiation in Multidrug-Resistant Gram-Negative Pathogenic Bacteria by a Synthetic Peptidomimetic’, *ACS infectious diseases*, 7(8), pp. 2152–2163.
54. Morel, C. *et al.* (2003) ‘Isoflavones as potentiators of antibacterial activity’, *Journal of agricultural and food chemistry*, 51(19), pp. 5677–5679.
55. Njimoh, D.L. *et al.* (2015) ‘Antimicrobial Activities of a Plethora of Medicinal Plant Extracts and Hydrolates against Human Pathogens and Their Potential to Reverse Antibiotic Resistance’, *International journal of microbiology*, 2015, p. 547156.
56. Oluwatuyi, M., Kaatz, G.W. and Gibbons, S. (2004) ‘Antibacterial and resistance modifying activity of Rosmarinus officinalis’, *Phytochemistry*, 65(24), pp. 3249–3254.
57. Penwell, W.F. *et al.* (2015) ‘Molecular mechanisms of sulbactam antibacterial activity and resistance determinants in Acinetobacter baumannii’, *Antimicrobial agents and chemotherapy*, 59(3), pp. 1680–1689.
58. Ponnusamy, K. *et al.* (2010) ‘Indirubin potentiates ciprofloxacin activity in the NorA efflux pump of Staphylococcus aureus’, *Scandinavian journal of infectious diseases*, 42(6-7), pp. 500–505.
59. Ferraz, Carlos A N et al. (2020) “Potentiation of antibiotic activity by chalcone (E)-1-(4'-aminophenyl)-3-(furan-2-yl)-prop-2-en-1-one against gram-positive and gram-negative MDR strains.” *Microbial pathogenesis* vol. 148, p. 104453.
60. Sharma, S. *et al.* (2010) ‘Piperine as an inhibitor of Rv1258c, a putative multidrug efflux pump of Mycobacterium tuberculosis’, *The Journal of antimicrobial chemotherapy*, 65(8), pp. 1694–1701.

61. Shiu, W.K.P. *et al.* (2013) ‘A new plant-derived antibacterial is an inhibitor of efflux pumps in Staphylococcus aureus’, *International journal of antimicrobial agents*, 42(6), pp. 513–518.
62. da Silva, M.M.C. *et al.* (2021) ‘Potentiation of Antibiotic Activity by a Meldrum’s Acid Arylamino Methylene Derivative against Multidrug-Resistant Bacterial Strains’, *Indian journal of microbiology*, 61(1), pp. 100–103.
63. Stavri, M., Piddock, L.J.V. and Gibbons, S. (2007) ‘Bacterial efflux pump inhibitors from natural sources’, *The Journal of antimicrobial chemotherapy*, 59(6), pp. 1247–1260.
64. Stermitz, F.R. *et al.* (2000) ‘Synergy in a medicinal plant: antimicrobial action of berberine potentiated by 5’-methoxyhydnocarpin, a multidrug pump inhibitor’, *Proceedings of the National Academy of Sciences of the United States of America*, 97(4), pp. 1433–1437.
65. Stogios, P.J. *et al.* (2013) ‘Structure-guided optimization of protein kinase inhibitors reverses aminoglycoside antibiotic resistance’, *Biochemical Journal*, 454(2), pp. 191–200.
66. Suga, T. *et al.* (2012) ‘Aranorosin circumvents arbekacin-resistance in MRSA by inhibiting the bifunctional enzyme AAC(6’)/APH(2″)’, *The Journal of antibiotics*, 65(10), pp. 527–529.
67. Thorarensen, A. *et al.* (2001) ‘3-Arylpiperidines as potentiators of existing antibacterial agents’, *Bioorganic & medicinal chemistry letters*, 11(14), pp. 1903–1906.
68. Upadhyay, H.C. *et al.* (2012) ‘Bioenhancing and antimycobacterial agents from Ammannia multiflora’, *Planta medica*, 78(1), pp. 79–81.
69. Vaara, M. (2009) ‘New approaches in peptide antibiotics’, *Current opinion in pharmacology*, 9(5), pp. 571–576.
70. Vaara, M. *et al.* (2010) ‘A novel polymyxin derivative that lacks the fatty acid tail and carries only three positive charges has strong synergism with agents excluded by the intact outer membrane’, *Antimicrobial agents and chemotherapy*, 54(8), pp. 3341–3346.

71. Vaara, M. and Vaara, T. (1983) ‘Sensitization of Gram-negative bacteria to antibiotics and complement by a nontoxic oligopeptide’, *Nature*, 303(5917), pp. 526–528.
72. Vargiu, A.V. *et al.* (2014) ‘Molecular Mechanism of MBX2319 Inhibition of Escherichia coli AcrB Multidrug Efflux Pump and Comparison with Other Inhibitors’, *Antimicrobial Agents and Chemotherapy*, pp. 6224–6234. doi:10.1128/aac.03283-14.
73. Vargiu, A.V. and Nikaido, H. (2012) ‘Multidrug binding properties of the AcrB efflux pump characterized by molecular dynamics simulations’, *Proceedings of the National Academy of Sciences of the United States of America*, 109(50), pp. 20637–20642.
74. Wang, D. *et al.* (2018) ‘Inhibitory effects of silybin on the efflux pump of methicillin-resistant Staphylococcus aureus’, *Molecular medicine reports*, 18(1), pp. 827–833.
75. Yuan, Y. *et al.* (2021) ‘Evaluation of Heterocyclic Carboxamides as Potential Efflux Pump Inhibitors in’, *Antibiotics (Basel, Switzerland)*, 11(1). doi:10.3390/antibiotics11010030.
76. Zhang, J. *et al.* (2019) ‘Insights on the Multifunctional Activities of Magnolol’, *BioMed research international*, 2019, p. 1847130.
